# Efficient helical columnar emitters of chiral homoleptic Pt(ii) metallomesogens for circularly polarized electroluminescence†

**DOI:** 10.1039/d4sc05781c

**Published:** 2024-10-10

**Authors:** Guo Zou, Zhenhao Jiang, Dong Li, Qihuan Li, Yixiang Cheng

**Affiliations:** a State Key Laboratory of Analytical Chemistry for Life Science, School of Chemistry and Chemical Engineering, Nanjing University Nanjing 210023 P. R. China yxcheng@nju.edu.cn

## Abstract

Chiral organometallic Pt(ii) complexes have been demonstrated to be excellent circularly polarized luminescence (CPL) materials due to their rich phosphorescence and strong self-assembly characteristics. However, it remains a formidable task to simultaneously achieve high luminance (*L*) and electroluminescence dissymmetry factor (*g*_EL_) values for circularly polarized electroluminescence (CP-EL) devices of Pt(ii) complex-based emitters. In this study, we carry out a straightforward and efficient protocol to construct highly CPL-active helical columnar (
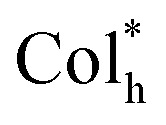
) emitters by using chiral homoleptic triazolatoplatinum(ii) metallomesogens (*R*/*S*-HPt). The peripheral flexible groups can not only improve solubility but also favor the induction of chirality and liquid crystal behavior. The resultant complexes *R*/*S*-HPt can self-assemble into the 
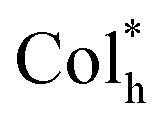
 mesophase over a broad temperature range (6–358 °C) and exhibit excellent phosphorescence (*Φ*: up to 86%), resulting in intense CPL signals after thermal annealing (*λ*_em_ = 615 nm and |*g*_em_| = 0.051). Using emitting layers (EML) based on *R*/*S*-HPt in solution-processed CP-EL devices, *L*_max_ and |*g*_EL_| of CP-EL can reach up to 11 379 cd m^−2^ and 0.014, respectively. With comprehensive consideration of *L*_max_ and *g*_EL_, this investigation shows the excellent performances among Pt(ii) complex-based CP-EL devices.

## Abstract 2

Helical columnar () emitters of homoleptic platinum(ii) metallomesogens (*R*/*S*-HPt) are constructed *via* chiral self-assembly, and can be applied to realize efficient solution-processed circularly polarized electroluminescence (CP-EL) devices.

## Graphical abstract

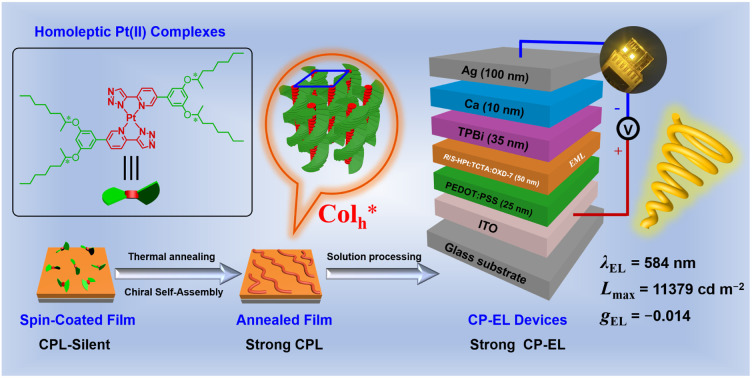

## Introduction

Devoting major efforts to develop efficient CP-EL devices is highly meaningful, because they can not only emit circularly polarized light directly but also have practical applications, such as optical data storage,^1^ optical spintronics^2^ and 3D displays.^3^ To date, increasing chiral emitters have been constructed and employed in EML for CP-EL devices, including chiral organometallic complexes,^4^ chiral polymers,^5^ chiral small organic molecules^6^ and chiral supramolecular assemblies.^7^ On the other hand, four-coordinate square-planar organometallic d^8^ Pt(ii) complexes have garnered increasing attention due to their intriguing and rich transition states.^4*b*,8^ Currently, aside from the rare chiral tetradentate Pt(ii) complexes,^9^ most reported chiral Pt(ii) complexes are heteroleptic, which can be generally categorized into bidentate,^4*c*,10^ and tridentate^4*a*,*b*,11^ Pt(ii) complexes, with various chirality sources of point,^12^ axial,^4*c*^ planar^4*b*^ and helical^10*b*^ chiral architectures. These complexes often exhibit strong circularly polarized photoluminescence (CP-PL) introduced by chiral ligands,^4*b*,*c*,10*b*,*c*,11*a*,13^ a supramolecular matrix with chiral reagents,^14^ or spontaneous symmetry breaking.^15^ However, the potential and practical applications of Pt(ii) complex-based CP-EL devices remain limited due to their unsatisfactory CP-EL performances (*L*_max_ < 10 000 cd m^−2^ and/or |*g*_EL_| < 10^−2^).^4*a*–*c*,10*b*,*c*,11*a*,13,16^ Notably, compared to chiral heteroleptic Pt(ii) complexes, investigations into chiral homoleptic Pt(ii) complexes have never been reported. Nonetheless, we recognize that homoleptic Pt(ii) complexes can serve as emitters to realize highly efficient organic light-emitting devices (OLEDs). For instance, Gnade and co-workers utilized pyridylpyrazolato-based homoleptic Pt(ii) complexes to fabricate evaporated OLEDs, which have an external quantum efficiency (EQE) of 31.1% and a *L*_max_ of up to circa 11 000 cd m^−1^.^17^ Subsequently, Chi *et al.* also confirmed that the maximum EQE and *L*_max_ can be measured to be 27.4% and more than 15 000 cd m^−1^, respectively, by isoquinolinylpyrazolato-functionalized homoleptic Pt(ii) complexes.^18^ Therefore, we speculate that homoleptic Pt(ii) complexes hold promise as luminophores for designing and constructing chiral emitters to fabricate high-performance CP-EL devices.

As one of the most significant soft materials, liquid crystals (LCs) exhibit high sensitivity to chiral group perturbations and demonstrate long-range order in their condensed state, thus playing a crucial role in chiral assemblies and CP-EL devices.^10*c*,19^ Chiral LCs have been widely utilized to effectively enhance the *g*_EL_ values of CP-EL-active materials, due to the formation of regular helical and periodic superstructures during the chiral assembly process.^20^ Chiral Pt(ii) metallomesogens (metal-containing liquid crystals), combine highly efficient phosphorescence with excellent assembly capacity, leading to numerous interesting investigations in chiral co-assemblies^14^ and CP-EL materials.^10*c*,16*b*^ Very recently, we demonstrated that remarkable CPL signals (*λ*_em_ = 642 nm and |*g*_em_| = 0.27) of achiral nematic Pt(ii) metallomesogens can be realized through the co-assembly strategy with anchored binaphthyl-containing chiral inducers.^14^ Wang and co-workers developed CP-EL devices based on point-chiral Pt(ii) metallomesogens, achieving an EQE, *L*_max_ and *g*_EL_ value of up to 11.3%, 7150 cd m^−2^ and 10^−2^, respectively.^10*c*^ However, it remains a formidable challenge to simultaneously achieve high luminance and high *g*_EL_ values for CP-EL devices of Pt(ii) complexes. Moreover, compared to Pt(ii) metallomesogens with a cholesteric (N*-LCs), chiral smectic (SmC*/SmA*) and twist grain boundary (TGB*) mesophase, the chiral columnar mesophase of Pt(ii) metallomesogens is less studied.^12,14,15^

**Scheme 1:**
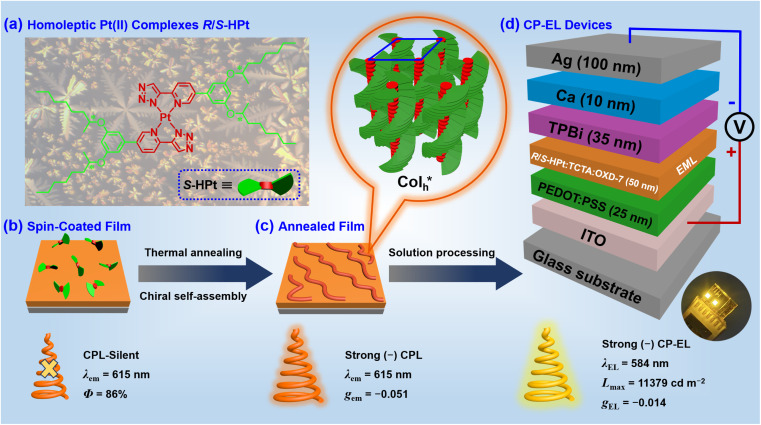
(a) Chemical structures of homoleptic Pt(ii) complexes *R*/*S*-HPt; (b) schematic diagram of the possible initial irregular accumulation of *S*-HPt; (c) schematic diagram of the possible helical chiral self-assembly of *S*-HPt after thermal annealing treatment; (d) device configuration of the CP-EL devices.

In order to construct new types of efficient CP-EL emitters, we have designed and synthesized a pair of enantiomeric homoleptic Pt(ii) metallomesogens (*R*/*S*-HPt) with pyridyltriazolato that features point chirality as cyclometalated ligands (Scheme 1a). The unannealed films of *R*/*S*-HPt exhibit high emission (*λ*_em_ = 615 nm and *Φ*: up to 86%) but are silent in CPL (Scheme 1b). Interestingly, strong CPL signals (*λ*_em_ = 615 nm and |*g*_em_| = 0.051) can be realized *via* constructing the 
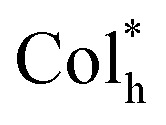
 mesophase after thermal annealing (Scheme 1c). Importantly, both chiral Pt(ii) complexes were utilized in EML to fabricate solution-processed CP-EL devices, achieving simultaneously high luminance and high *g*_EL_ values (Scheme 1d, *L*_max_ = 11 379 cd m^−2^ and |*g*_EL_| = 0.014).

## Results and discussion

### Synthesis and characterization

The chiral homoleptic Pt(ii) metallomesogens *R*/*S*-HPt were synthesized in high yields (93% for *R*-HPt and 88% for *S*-HPt) *via* the reaction of K_2_PtCl_4_ with chiral *N*^*N*-cyclometalated ligands *R*/*S*-7 (Scheme S1†). These ligands were prepared using a series of Sonogashira^21^ and Sharpless copper-catalyzed Huisgen 1,3-dipolar cycloaddition protocols, *etc.*^22^ This approach eliminates the need for the redundant synthesis of chloride-containing organoplatinum(ii) precursors.^23^ Detailed synthesis and characterization data, including ^1^H and ^13^C NMR, chiral HPLC, and MALDI-TOF MS data, are provided in the ESI.† The targeted chiral Pt(ii) complexes were further purified *via* triple recrystallization in a mixture of dichloromethane and methanol, followed by column chromatography before investigation.

### Photophysical properties and computational investigation

**Fig. 1:**
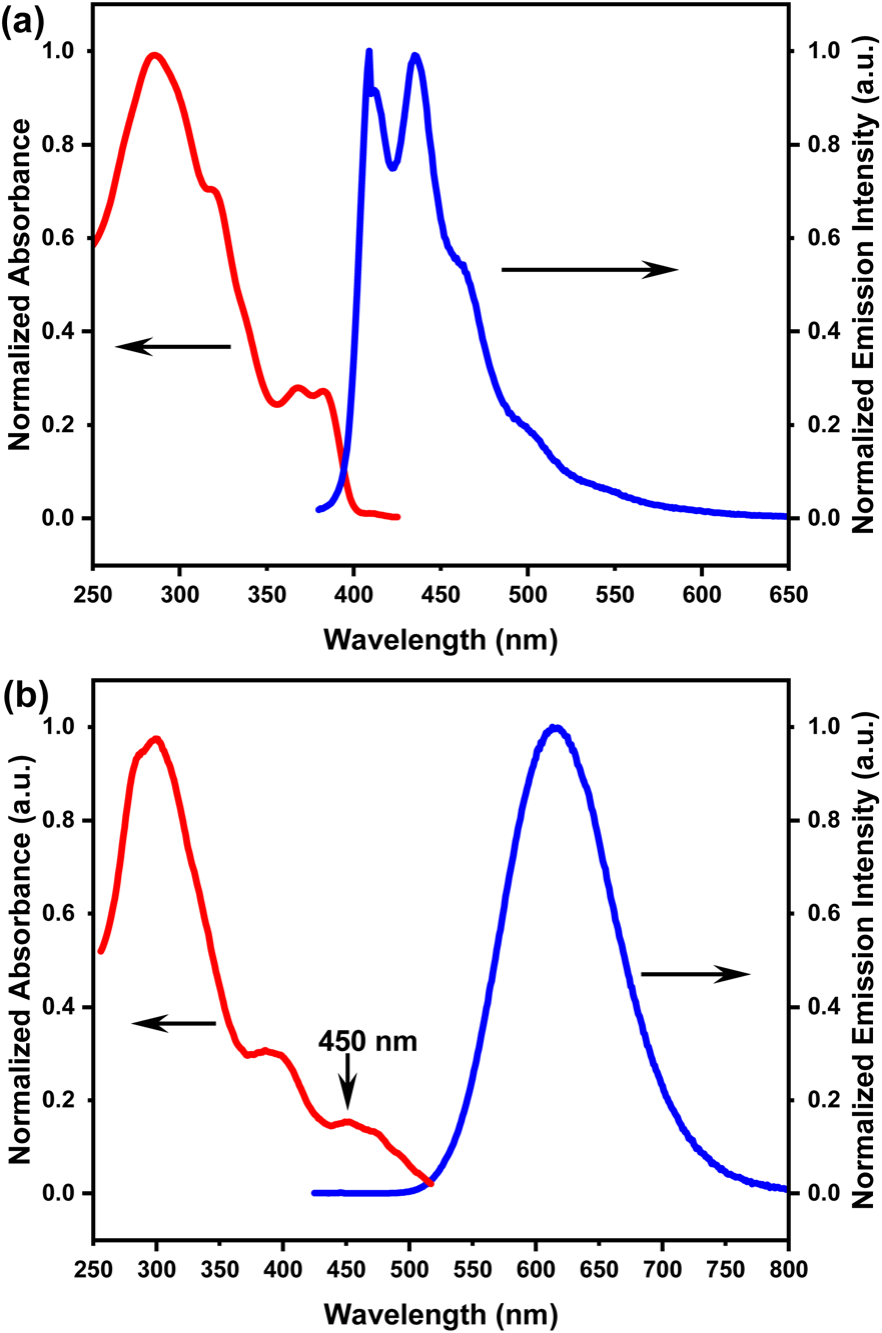
Photophysical spectra of homoleptic Pt(ii) complex *R*-HPt in (a) chloroform solution (1.0 × 10^−5^ mol L^−1^) and (b) spin-coated films.

Table 1 shows the ultraviolet-visible (UV-vis) absorption and photoluminescence (PL) emission data of homoleptic Pt(ii) complexes *R*/*S*-HPt in solution and spin-coated films. In dilute chloroform solutions, *R*/*S*-HPt exhibit two main UV-vis absorption bands (Fig. 1a and S1a†). The high-energy UV-vis bands (≤350 nm) appeared at circa *λ* = 285 nm and 320 nm, deriving from a typical ligand-centered π → π* transition, as confirmed by comparing with the UV-vis spectra of their corresponding free ligands (Fig. S1c†). The low-energy absorption regions are centered at 368 nm and 383 nm with relatively low molar extinction coefficients, which can be attributed to transitions involving metal-to-ligand charge transfer (MLCT) and intraligand charge transfer (ILCT) to a certain extent, as corroborated by TDDFT results (see below).^24^ In spin-coated films, three distinct absorption bands were identified for *R*/*S*-HPt under ambient conditions (Fig. 1b and S1b†). The ligand-centered and MLCT/ILCT bands are preserved compared to those in solutions, exhibiting slight red shifts of 14 nm and 12 nm, respectively. Notably, a newly generated broad absorption band centered at approximately 450 nm was observed, stemming from metal-metal-to-ligand charge transfer (MMLCT) transitions, indicative of intermolecular Pt⋯Pt and π–π stacking interactions in the condensed state.^25^

**Table 1:** Photophysical data of homoleptic Pt(ii) complexes in chloroform solution and spin-coated films at room temperature

Complexes	Absorption (nm) [*ε*/10^4^ × (dm^3^ mol^−1^ cm^−1^)]				Emission (nm)		*Φ* a (%)	*Φ* b (%)	*τ* a (ns)	*τ* b (μs)
					Solution	Film				
*R*-HPt	285 (5.43)	320 (3.87)	368 (1.59)	383 (1.53)	411, 435, 465, 501	615	1	83	1.65	10.5
*S*-HPt	285 (5.23)	320 (3.79)	368 (1.55)	383 (1.52)	411, 435, 465, 501	615	2	86	1.64	11.3

a In degassed chloroform solution (*M* = 1.0 × 10^−5^ mol L^−1^).

b In a spin-coated film. Excitation wavelength = 365 nm.

**Fig. 2:**
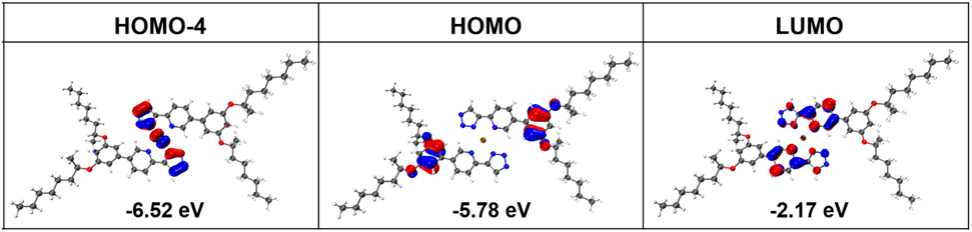
Frontier molecular orbitals and energy level of *R*-HPt.

To elucidate the properties of the excited states and charge transfer transitions, density functional theory (DFT) and time-dependent DFT (TDDFT) calculations for the homoleptic Pt(ii) complexes *R*/*S*-HPt were carried out using the Gaussian 16 program package (see details in the ESI†).^26^ In chloroform, the computational absorption spectral profiles of homoleptic Pt(ii) complexes closely match the experimental results (Fig. S2 and S3†). The predicted absorption spectra indicate that the low-energy absorption bands are mainly responsible by the S_1_, S_2_ and S_3_ excitations. The oscillator strength of the S_3_ excitation (*f* = 0.2889) is significantly higher than those of the S_1_ (*f* = 0.0095) and S_2_ (*f* = 0.0029) excitations. This result suggests that the S_3_ excitation plays an important role in the excited-state character of *R*/*S*-HPt, indicating that the low-energy absorption bands are dominated by transitions from the highest occupied molecular orbital (HOMO) → lowest unoccupied molecular orbital (LUMO), HOMO−1 → LUMO, HOMO−2 → LUMO and HOMO−4 → LUMO. Combining their frontier molecular orbitals, excitation energies and charge transfer transitions (Fig. 2, S4, Tables S1 and S2†), the low-energy absorption bands can be ascribed to a mixture of MLCT and ILCT from triazolyl and phenyl segments to pyridine rings.^27^

The PL spectra of the representative complex *S*-HPt at various concentrations were recorded at room temperature (Fig. S5†). At low concentrations (10^−6^ to 10^−5^ mol L^−1^), *S*-HPt exhibits monomeric emission at 411 nm and 435 nm with the lifetimes of 1.64–1.77 ns (Fig. S6 and S7†), originating from the ^1^MLCT transition. As the concentrations increased to 10^−4^ to 10^−3^ mol L^−1^, in addition to the monomeric emission bands (*λ* = 411 nm and 435 nm; *τ* = 2.85–3.87 ns, Fig. S8†), red-shifted excimer emissions were observed at 509 nm, 545 nm and 591 nm (*τ* = 10.29–34.68 μs, Fig. S9 and S10†), suggestive of concentration-dependent emission behavior and incomplete self-assembly of *S*-HPt, involving intermolecular Pt⋯Pt and π–π stacking interactions. Furthermore, the enhanced aggregation-state emissions of *R*/*S*-HPt were investigated in spin-coated films (Fig. 1b and S1b†), showing highly intense orange-red emissions with almost identical peak wavelengths (*λ* = 615 nm). The ultra-high photoluminescence quantum yields of 83% for *R*-HPt and 86% for *S*-HPt can be attributed to their effective energy utilization. The long lifetimes of *R*-HPt (10.5 μs) and *S*-HPt (11.3 μs) are also associated with MMLCT emission processes and phosphorescence (Fig. S11†). The red shift of the excimer and/or aggregate emissions in doped films (547 nm for 20 wt%, 575 nm for 50 wt%, 590 nm for 80 wt% and 615 nm for 100 wt%, Fig. S12†) is due to enhanced overlap or stacking of the planar *R*/*S*-HPt molecules with increasing doping concentration, which is well consistent with previous reports.^17,24,28^

### Thermal properties and mesophase behavior

**Fig. 3:**
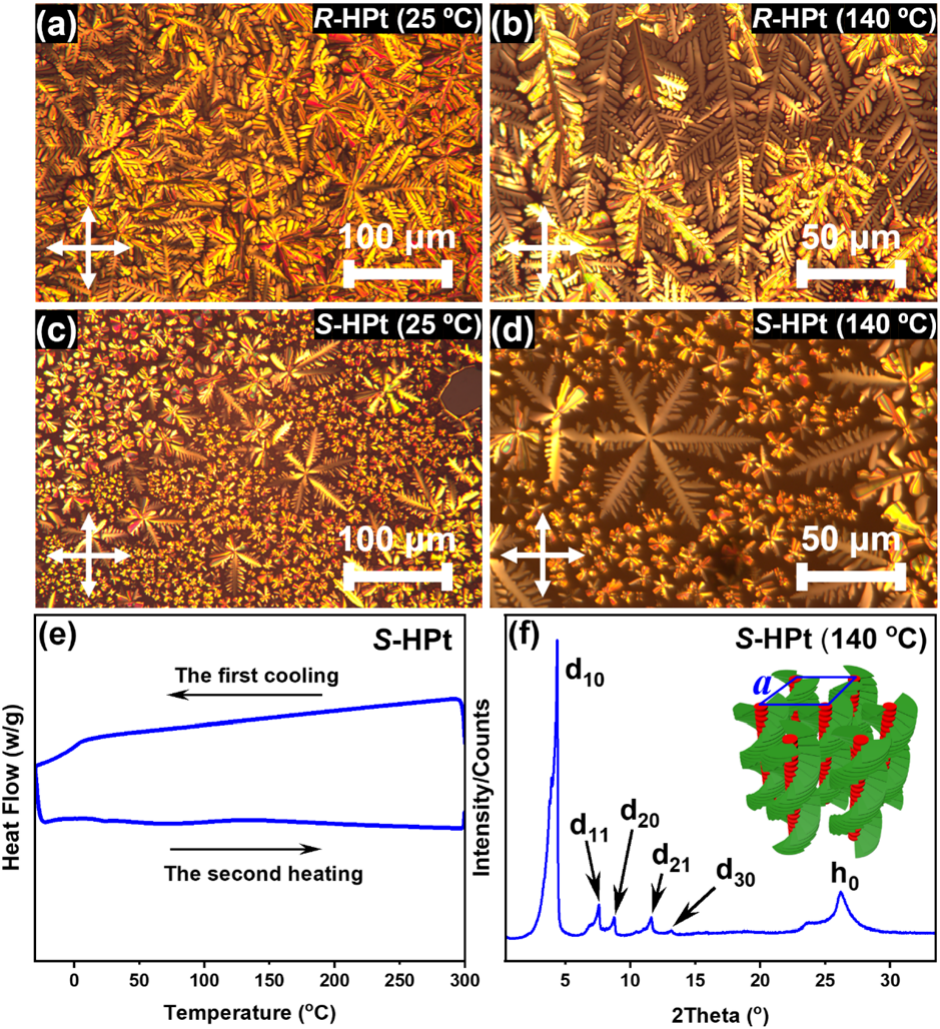
POM images of *R*-HPt (a and b) and *S*-HPt (c and d) on cooling; DSC curves (e) and XRD profile (f) of *S*-HPt.

The two enantiomeric homoleptic Pt(ii) complexes exhibit good thermal stability, as evidenced by thermogravimetric analysis (TGA). The degradation temperatures, corresponding to 5% weight loss, are both more than 360 °C for *R*/*S*-HPt (Fig. S13†). In polarizing optical microscopy (POM) measurements, *R*/*S*-HPt show a distinct birefringent phenomenon during both heating and cooling processes. Specifically, dendritic and snowflake-shaped textures were clearly observed (Fig. 3a–d), suggestive of the enantiotropic hexagonal columnar mesophase. Notably, the clear point temperatures of *R*/*S*-HPt are both as high as 358 °C (Fig. S14†), closely aligning with their degradation temperatures, indicating that these homoleptic Pt(ii) complexes possess ultra-high thermally stable mesophase structures. Furthermore, the liquid crystal states of *R*/*S*-HPt persist throughout a broad temperature range from isotropic fluid to room temperature, suggesting their potential as an ideal chiral self-assembly matrix for the fabrication and operation of CP-EL devices. Differential scanning calorimetry (DSC) experiments were subsequently performed on *R*/*S*-HPt over a temperature range of −30 °C to 300 °C (Fig. S15† and 3e). No obvious endothermic/exothermic peaks were observed throughout the experiments, possibly due to minor decomposition at elevated temperatures; a similar situation was also noted in previously reported homoleptic Pt(ii) complexes.^25,29^ As revealed from the cooling scan, *R*/*S*-HPt exhibited a glass transition and maintained the mesophase even down to 6 °C, indicating that both homoleptic complexes *R*/*S*-HPt are excellent room-temperature liquid crystals.

Variable temperature X-ray diffraction (XRD) was carried out to confirm the results revealed by POM and DSC. *S*-HPt was here selected as an example to elaborate their long-range order structures in LC states. Considering the broad mesophase range of *S*-HPt, three sets of temperature (25 °C, 140 °C and 280 °C) on cooling were selected for XRD measurements. As depicted in Fig. 3f, five characteristic diffractions were observed in the small-angle region at 140 °C with a 2*θ* of 4.38°, 7.59°, 8.75°, 11.63° and 13.14°. The pertinent *d* spacings were calculated to be 20.19 Å, 11.65 Å, 10.11 Å, 7.63 Å and 6.75 Å, respectively, which are well consistent with the ratio of 1 : 1/√3 : 1/2 : 1/√7 : 1/3. These peaks can be indexed as d_10_, d_11_, d_20_, d_21_ and d_30_ reflections. Whereas its chiroptical properties (see below), we tentatively speculate that *S*-HPt exhibits helical hexagonal columnar (
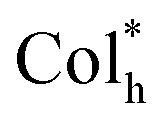
) mesophase structures with a lattice constant (*a*) of 23.33 Å. In addition, a broad diffuse peak (*h*_0_) of *d* = 3.41 Å was observed at 2*θ* = 26.19°, which is assigned to the intermolecular π–π stacking interactions intracolumn. Furthermore, the XRD curves and corresponding data at 25 °C and 280 °C are also recorded in Fig. S16 and Table S3,† showing parameters similar to those of *S*-HPt at 140 °C, although slight disturbances were noted at different temperatures. As expected, the XRD results of *R*-HPt (Fig. S17 and Table S3†) are well consistent with those of *S*-HPt. Based on the results of POM, DSC and XRD, we demonstrate that the homoleptic Pt(ii) complexes *R*/*S*-HPt are capable of forming the 
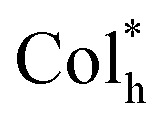
 mesophase with a broad temperature range and ultra-high thermal stability, which is essential for potential applications of high-performance CP-EL devices.

### Chiroptical properties

**Fig. 4:**
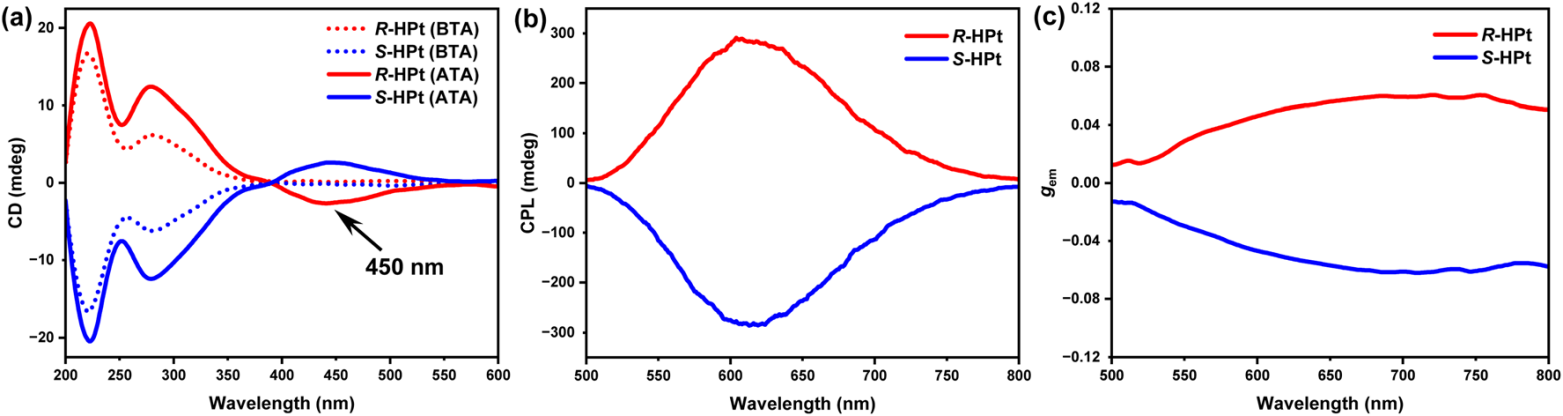
Chiroptical spectra of spin-coated films *R*/*S*-HPt: (a) CD spectra before thermal annealing (BTA, dashed line) and after thermal annealing (ATA, solid line); (b) CPL spectra and (c) *g*_em_ values *vs.* wavelength after thermal annealing at 140 °C.

Circular dichroism (CD) measurements were initially employed to probe the macroscopical chiroptical activity of the enantiomeric Pt(ii) complexes. As shown in Fig. 4a, the unannealed films *R*/*S*-HPt exhibit perfect mirror-image CD signals (*λ* ≤ 400 nm), featuring two distinct Cotton peaks of 220 (|*g*_abs_| = 1.89 × 10^−3^) and 280 nm (|*g*_abs_| = 1.16 × 10^−3^) within the ligand-centered and MLCT/ILCT transition regions. After thermal annealing at 140 °C (Fig. 4a), in addition to the initial Cotton effect signals of the unannealed films, newly generated mirror-image Cotton effect peaks were observed in the ^3^MMLCT transition range (*λ* = 450 nm and |*g*_abs_| = 6.82 × 10^−3^), indicative of the chiral self-assembly of homoleptic Pt(ii) complexes through intermolecular Pt⋯Pt and π–π stacking interactions at the LC state. To reveal the excited state properties of chirality, CPL experiments were carried out on the spin-coated films of the two enantiomeric Pt(ii) complexes. No obvious CPL signals were detected in the unannealed *R*/*S*-HPt films within the range of 500–800 nm (Fig. S18†). Notably, after annealing at 140 °C (Fig. 4b and c), the self-assembled films exhibited strong orange-red CPL signals (*λ*_em_ = 615 nm and |*g*_em_| = 0.051) with a clear mirror-image relationship, originating from the ^3^MMLCT transition and a chiral coupling environment for excited molecules.^15,30^

### Morphology study

**Fig. 5:**
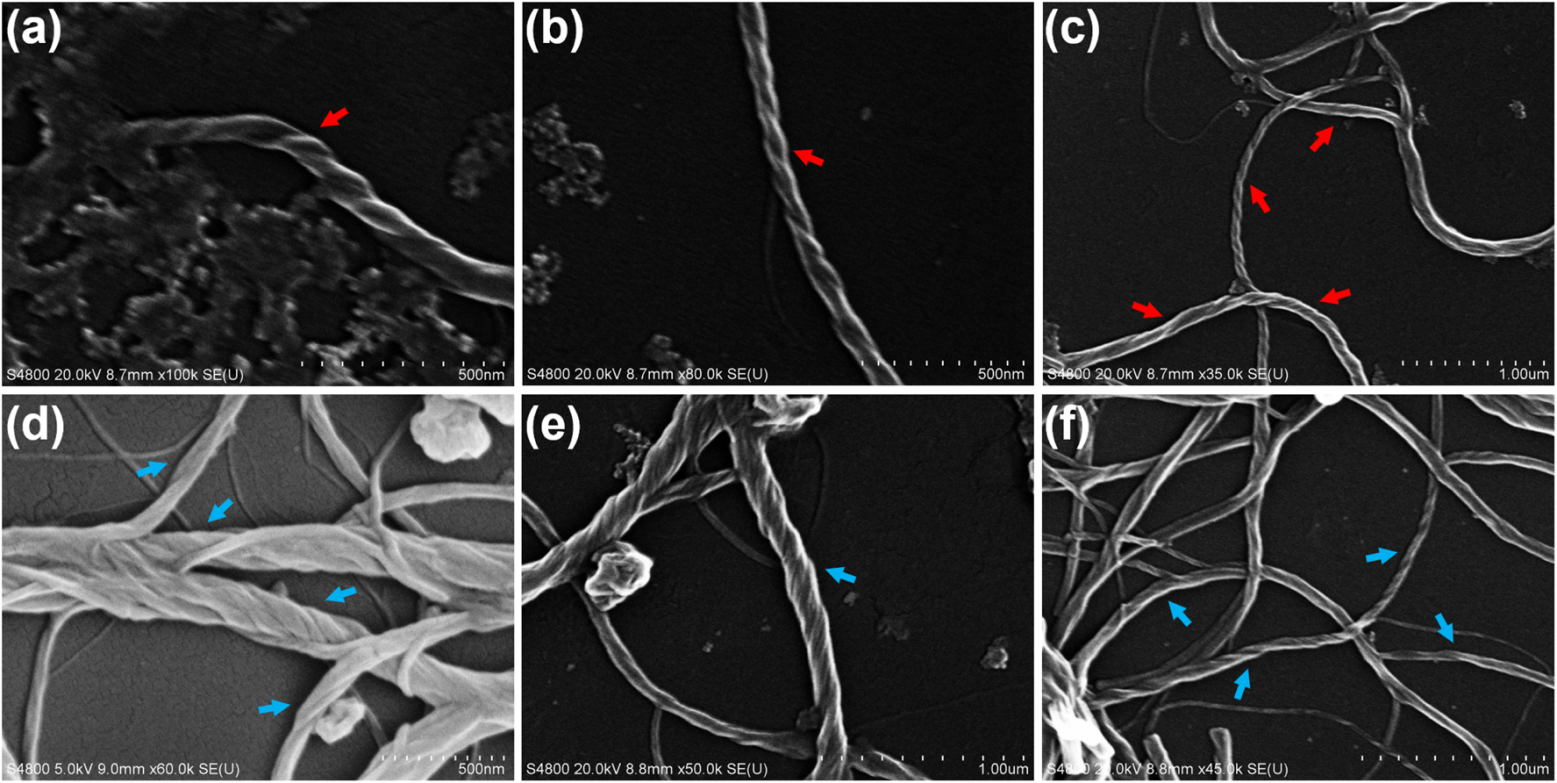
SEM images of chiral self-assemblies *R*-HPt (a–c) and *S*-HPt (d–f) after thermal annealing at 140 °C. (a) and (d): film, 1.0 × 10^−3^ mol L^−1^ in chloroform; (b) and (e): film, 1.0 × 10^−4^ mol L^−1^ in chloroform; (c) and (f): aggregated state, 1.0 × 10^−4^ mol L^−1^ in THF/H_2_O = 80/20 (v/v).

In order to investigate chiral self-assembled morphologies of homoleptic Pt(ii) complexes *R*/*S*-HPt, scanning electron microscopy (SEM) experiments were executed at 1.0 × 10^−3^ and 1.0 × 10^−4^ mol L^−1^ respectively in films and the aggregated state (1.0 × 10^−4^ mol L^−1^ in THF/H_2_O = 80/20, v/v). Before thermal annealing treatment, drop-coated films of *R*/*S*-HPt exhibited irregular accumulation (Fig. S19a and d†) and randomly distributed crystals (Fig. S19b and e†). The unannealed aggregates *R*/*S*-HPt formed globular structures with a regular shape and uniform size (Fig. S19c and f†). Interestingly, distinct nanofibers of *R*/*S*-HPt were observed not only in the films but also in the aggregated state after thermal annealing at 140 °C (Fig. 5a–c for *R*-HPt and Fig. 5d–f for *S*-HPt). The left- and right-handed helices were respectively observed for *R*-HPt and *S*-HPt, indicating a highly ordered helical molecular arrangement. Combining POM and XRD analyses, we demonstrate that homoleptic Pt(ii) complexes can form the 
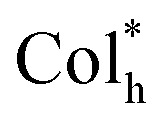
 mesophase *via* intermolecular Pt⋯Pt and π–π stacking interactions after thermal annealing treatment, resulting in the generation of intense CPL signals.

### Mechanism of chiral self-assembly

**Fig. 6:**
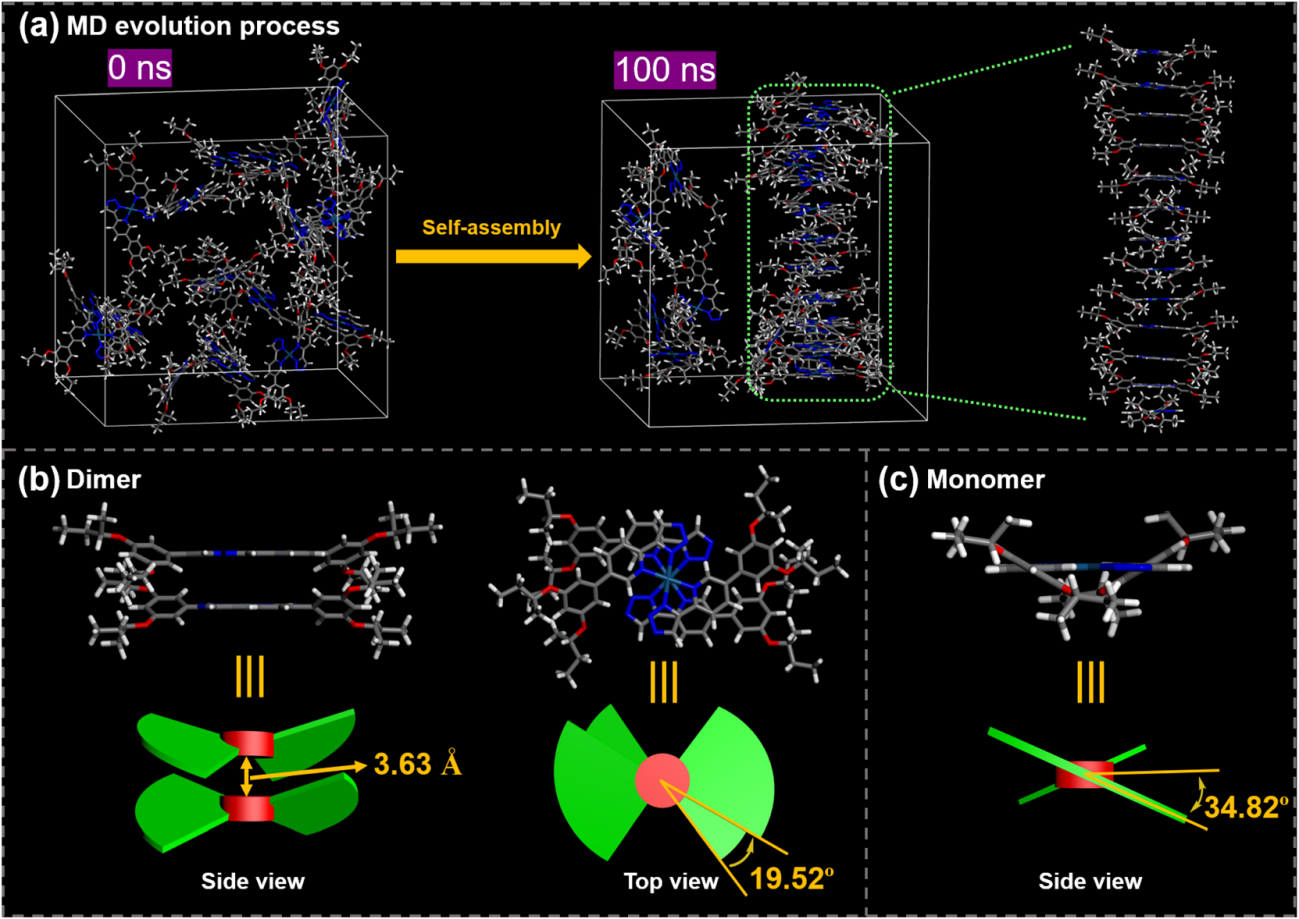
MD evolution process (a), the extracted dimer (b) and the monomer (c) of *S*-HPt-C4.

Molecular dynamics (MD) simulations were performed to deeply explore the interaction mechanisms involved in the chiral self-assembly process. To simplify the model corresponding to *S*-HPt, we employed *S*-HPt-C4, where the chiral OC_8_H_17_ side chains were replaced with shorter chiral OC_4_H_9_ chains, thereby reducing the number of atoms and computational costs. Details of the MD simulation methods are provided in the ESI.† As the equilibrium time increases, the molecules gradually aggregate together from an initially dispersed state. Prior to 100 ns of equilibrium time, *S*-HPt-C4 shows an ordered helical columnar arrangement at 140 °C (Fig. 6a). The stacking dimer of self-assemblies *S*-HPt-C4 was further extracted, demonstrating that the interplanar distance between adjacent Pt(ii)-coordinate planes is 3.63 Å (Fig. 6b), which is in good agreement with the earlier XRD analyses (Fig. 3f). In this helical column arrangement, adjacent stacked molecules are rotated by 19.52° along the stacking axis (Fig. 6b). Notably, *S*-HPt-C4 exhibits a nonplanar geometry between the phenyl group and pyridine ring to minimize the steric hindrance.^31^ The dihedral angle between the peripheral phenyl group and central Pt(ii)-coordinate plane of *S*-HPt-C4 is 34.82° (Fig. 6c). This rotation skeleton confers the molecule with a two-blade equidistant propeller-like geometry that effectively self-assembles into a right-handed helical column, which is supported by SEM experiments (Fig. 5d–f). It was also observed that the total energy of self-assemblies *S*-HPt-C4 is approximately −415 eV (Fig. S20a†) and the intermolecular binding energy was calculated to be −32.29 kcal mol^−1^ (Fig. S20b†). These results indicate that this type of homoleptic Pt(ii) complex can easily self-assemble as a stable helical columnar emitter *via* intermolecular Pt⋯Pt and π–π stacking interactions after thermal annealing, thus promoting the generation and amplification effect of CPL signals.

### Electrochemical properties

Cyclic voltammetry (CV) measurements were carried out in degassed dichloromethane solution at room temperature to investigate the redox characters of *R*/*S*-HPt (Fig. S21 and Table S4†). The onset of the first oxidation potential and the edges of the UV-vis absorption spectra were used to calculate the energy levels of the HOMO and LUMO of the corresponding homoleptic Pt(ii) complexes. The energy levels of the HOMO and LUMO are −5.68 eV and −2.58 eV for *R*-HPt and −5.71 eV and −2.61 eV for *S*-HPt, respectively.

### CP-EL devices

**Fig. 7:**
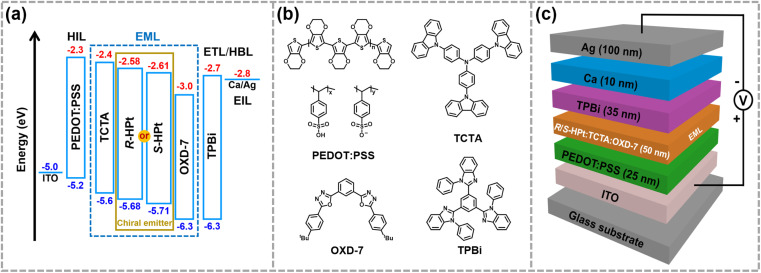
The energy level diagrams (a) and chemical structures (b) of related materials, and CP-EL device structure (c).

In view of the ultra-high thermal stability of the mesophase, strong phosphorescence, good solubility and novel 
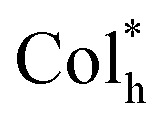
 superstructures, homoleptic Pt(ii) complexes were applied as emitters to fabricate low-cost solution-processed CP-EL devices. The hole-transport material 4,4′,4′′-tris(carbazol-9-yl)triphenylamine (TCTA) and the electron-transport material 1,3-bis(5-(4- (*tert*-butyl)phenyl)-1,3,4-oxadiazol-2-yl)benzene (OXD-7) have high triplet state energies (3.21–3.48 eV), and thus they were employed as host materials to ensure an efficient host–guest energy transfer and to facilitate balanced charge transport. Fig. 7a and b show the energy level diagrams and chemical structures of related materials. The HOMO and LUMO energy levels of the guests lie between the the HOMO energy level of OXD-7 (−6.3 eV) and the LUMO energy level of TCTA (−2.4 eV), indicative of a favorable match between the guest and the host materials. Solution processed CP-EL devices were fabricated with the configuration (Fig. 7c) of indium tin oxide glass (ITO)/poly(2,3-dihydrothieno-1,4-dioxin)-poly(styrenesulfonate) (PEDOT:PSS) (25 nm)/blended host materials: homoleptic Pt(ii) complex (50 nm)/1,3,5-tris(*N*-phenylbenz-imidazole-2-yl)-benzene (TPBi, 35 nm)/Ca (10 nm)/Ag (100 nm). PEDOT:PSS, TPBi and Ca act as the hole-injection layer (HIL), the electron-transporting layer (ETL) and the electron-injecting layer (EIL), respectively. The EML was fabricated by spin-coating a mixed chloroform solution of the guest (80 wt%) and the blended host of TCTA : OXD-7 (1 : 1).

**Fig. 8:**
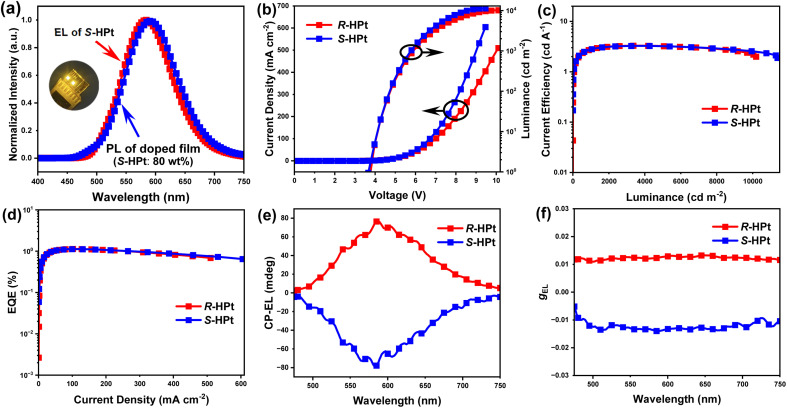
The EL and PL spectra of the *S*-HPt-based CP-EL device and doped film (a); current density and luminance as a function of voltage (b), the dependence of CE on the luminance (c), EQE *vs.* current density (d), and CP-EL spectra (e) and *g*_EL_ values (f) of *R*/*S*-HPt-based CP-EL devices (inset: photo of the *S*-HPt-based CP-EL device during working).

The electroluminescence (EL) performances of homoleptic Pt(ii) complexes are depicted in Fig. 8, S22 and Table S5.† CP-EL devices *R*/*S*-HPt exhibit intense EL emissions (*λ*_EL_ = 584 nm) with the 1931 Commission Internationale de l’Eclairage (CIE) coordinates of (0.50, 0.49), which are virtually the same as the PL spectra of their doped films (Fig. 8a and S22a†). It is very reasonable that the two pure enantiomer Pt(ii) complexes *R*/*S*-HPt have identical EL spectra. Little change is found from the EL spectra of the device *S*-HPt at different driving voltages of 5–9 V (Fig. S22b†), indicative of its stable EL capabilities. Devices *R*/*S*-HPt exhibit high performance with a *V*_on_ of 3.7/3.6 V, *L*_max_ of 10 184/11 379 cd m^−2^, CE_max_ of 3.26/3.30 cd A^−1^ and EQE_max_ of 1.14/1.12%, respectively.

The CP-EL characters of homoleptic Pt(ii) complexes are depicted in Fig. 8e and f and Table S5.† The CP-EL spectra of *R*/*S*-HPt-based devices show a good mirror-image relationship within the range of 475–750 nm, with *g*_EL_ values at *λ*_EL_ of 0.012 and −0.014, respectively. The *g*_EL_ values are an order of magnitude higher than those of the majority of Pt(ii) complex-based CP-EL devices.^4*b*,*c*,11*a*,13,16^ With comprehensive consideration of *L*_max_ and *g*_EL_, these devices exhibit excellent performances among Pt(ii) complex-based CP-EL devices (Table S6†). Notably, the utilization of helical columnar emitters of Pt(ii) complexes in CP-EL devices is unprecedented.

## Conclusion

In the present work, we first demonstrated a straightforward and efficient protocol to realize helical columnar emitters *R*/*S*-HPt, which are triggered by intermolecular Pt⋯Pt and π–π stacking interactions within the self-assembly process after thermal annealing. The peripheral flexible groups can not only ensure good solubility and liquid crystal behaviors, but also promote chiral induction. *R*/*S*-HPt can self-assemble into an ultra-high thermally stable 
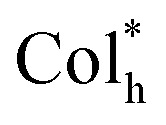
 mesophase (up to 358 °C) and exhibit strong phosphorescence (*Φ*: up to 86%), resulting in intense CPL signals (*λ*_em_ = 615 nm and |*g*_EL_| = 0.051). By utilizing *R*/*S*-HPt for chiral EML *via* solution processing methods, *L*_max_ and |*g*_EL_| of CP-EL devices can reach up to 11 379 cd m^−2^ and 0.014, respectively. Thus, this investigation provides an innovative platform for the design of efficient CPL materials and related applications.

## Author contributions

G. Z., Z.-H. J., D. L., and Q.-H. L. carried out most of the investigation and developed the methodology. G. Z. is in charge of writing – original draft with assistance from all authors. Y.-X. C. supervised the work and writing – review & editing, project administration and funding acquisition.

## Conflicts of interest

There are no conflicts to declare.

## Supplementary Material

SC-015-D4SC05781C-s001

## Data Availability

The data that support the findings of this study are available within the article and its ESI.†

## References

[cit1] Huck N. P. M., Jager W. F., de Lange B., Feringa B. L. (1996). Science.

[cit2] Farshchi R., Ramsteiner M., Herfort J., Tahraoui A., Grahn H. T. (2011). Appl. Phys. Lett..

[cit3] Zinna F., Giovanella U., Bari L. D. (2015). Adv. Mater..

[cit4] Han J., Lu H., Xu Y., Guo S., Zheng X., Tao P., Liu S., Zhang X., Zhao Q. (2020). J. Organomet. Chem..

[cit5] Geng Z., Zhang Y., Zhang Y., Quan Y., Cheng Y. (2022). Angew. Chem., Int. Ed..

[cit6] Shen Z., Wang T., Shi L., Tang Z., Liu M. (2015). Chem. Sci..

[cit7] Zhang Y., Li Y., Quan Y., Ye S., Cheng Y. (2023). Angew. Chem., Int. Ed..

[cit8] Baldo M. A., O'Brien D. F., You Y., Shoustikov A., Sibley S., Thompson M. E., Forrest S. R. (1998). Nature.

[cit9] Yuan L., Ding Q.-J., Tu Z.-L., Liao X.-J., Luo X.-F., Yan Z.-P., Wu Z.-G., Zheng Y.-X. (2022). Chin. Chem. Lett..

[cit10] Shen C., Anger E., Srebro M., Vanthuyne N., Deol K. K., Jefferson T. D., Muller G., Williams J. A. G., Toupet L., Roussel C., Autschbach J., Réau R., Crassous J. (2014). Chem. Sci..

[cit11] Han J., Wang Y., Wang J., Wu C., Zhang X., Yin X. (2022). J. Organomet. Chem..

[cit12] Yang B., Ni H., Wang H., Hu Y., Luo K., Yu W. (2020). J. Phys. Chem. C.

[cit13] Fu G., He Y., Li W., Wang B., Lü X., He H., Wong W.-Y. (2019). J. Mater. Chem. C.

[cit14] Li Q., Zou G., Li D., Liu C., Gao W., Li Y., Cheng Y. (2024). Adv. Opt. Mater..

[cit15] Yang B., Zou G., Zhang S., Ni H., Wang H., Xu W., Yang C., Zhang H., Yu W., Luo K. (2021). Angew. Chem., Int. Ed..

[cit16] Yan Z.-P., Luo X.-F., Liu W.-Q., Wu Z.-G., Liang X., Liao K., Wang Y., Zheng Y.-X., Zhou L., Zuo J.-L., Pan Y., Zhang H. (2019). Chem.–Eur. J..

[cit17] Wang Q., Oswald I. W. H., Yang X., Zhou G., Jia H., Qiao Q., Chen Y., Hoshikawa-Halbert J., Gnade B. E. (2014). Adv. Mater..

[cit18] Chen W.-C., Sukpattanacharoen C., Chan W.-H., Huang C.-C., Hsu H.-F., Shen D., Hung W.-Y., Kungwan N., Escudero D., Lee C.-S., Chi Y. (2020). Adv. Funct. Mater..

[cit19] Akagi K. (2009). Chem. Rev..

[cit20] Zhang Y., Yu W., Li H., Zheng W., Cheng Y. (2023). Chem.–Eur. J..

[cit21] Tuesuwan B., Kerwin S. M. (2006). Biochemistry.

[cit22] Loren J. C., Krasiński A., Fokin V. V., Sharpless K. B. (2005). Synlett.

[cit23] Gareth Williams J. A., Develay S., Rochester D. L., Murphy L. (2008). Coord. Chem. Rev..

[cit24] Chang S.-Y., Kavitha J., Li S.-W., Hsu C.-S., Chi Y., Yeh Y.-S., Chou P.-T., Lee G.-H., Carty A. J., Tao Y.-T., Chien C.-H. (2006). Inorg. Chem..

[cit25] Liao C.-T., Chen H.-H., Hsu H.-F., Poloek A., Yeh H.-H., Chi Y., Wang K.-W., Lai C.-H., Lee G.-H., Shih C.-W., Chou P.-T. (2011). Chem.–Eur. J..

[cit26] Frisch M. J. , Trucks G. W., Schlegel H. B., Scuseria G. E., Robb M. A., Cheeseman J. R., Scalmani G., Barone V., Petersson G. A., Nakatsuji H., Li X., Caricato M., Marenich A. V., Bloino J., Janesko B. G., Gomperts R., Mennucci B., Hratchian H. P., Ortiz J. V., Izmaylov A. F., Sonnenberg J. L., Williams D., Ding F., Lipparini F., Egidi F., Goings J., Peng B., Petrone A., Henderson T., Ranasinghe D., Zakrzewski V. G., Gao J., Rega N., Zheng G.; Liang W., Hada M., Ehara M., Toyota K., Fukuda R., Hasegawa J., Ishida M., Nakajima T., Honda Y., Kitao O., Nakai H., Vreven T., Throssell K., Montgomery Jr J. A., Peralta J. E., Ogliaro F., Bearpark M. J., Heyd J. J., Brothers E. N., Kudin K. N., Staroverov V. N., Keith T. A., Kobayashi R., Normand J., Raghavachari K., Rendell A. P., Burant J. C.; Iyengar S. S., Tomasi J., Cossi M., Millam J. M.; Klene M., Adamo C., Cammi R., Ochterski J. W., Martin R. L., Morokuma K., Farkas O., Foresman J. B. and Fox D. J., Gaussian 16, Revision B.01, Gaussian, Inc., Wallingford, CT, 2016

[cit27] Prabhath M. R. R., Romanova J., Curry R. J., Silva S. R. P., Jarowski P. D. (2015). Angew. Chem., Int. Ed..

[cit28] Brulatti P., Fattori V., Muzzioli S., Stagni S., Mazzeo P. P., Braga D., Maini L., Milita S., Cocchi M. (2013). J. Mater. Chem. C.

[cit29] Cuerva C., Campo J. A., Cano M., Lodeiro C. (2016). Chem.–Eur. J..

[cit30] San Jose B. A., Matsushita S., Akagi K. (2012). J. Am. Chem. Soc..

[cit31] Concellón A., Lu R.-Q., Yoshinaga K., Hsu H.-F., Swager T. M. (2021). J. Am. Chem. Soc..

