# Supplementary material for: Efficient helical columnar emitters of chiral homoleptic Pt(ii) metallomesogens for circularly polarized electroluminescence
Source: Chem Sci. 2024 Oct 10;15(44):18534–42. doi: 10.1039/d4sc05781c (PMC11488679; doi:10.1039/d4sc05781c)
Supplement: SC-015-D4SC05781C-s001 [file SC-015-D4SC05781C-s001.pdf]

Supplementary Information (SI) for Chemical Science.
This journal is © The Royal Society of Chemistry 2024

Electronic Supplementary Information for

Efficient Helical Columnar Emitters of Chiral Homoleptic Pt(II) Metallomesogens for Circularly Polarized Electroluminescence

*Guo Zou, Zhenhao Jiang, Dong Li, Qihuan Li and Yixiang Cheng**

State Key Laboratory of Analytical Chemistry for Life Science, School of Chemistry and Chemical Engineering, Nanjing University, Nanjing 210023, China
E-mail: yxcheng@nju.edu.cn

1. Experimental section

2. Synthetic procedures and characterization

3. UV–*vis* absorption and PL spectra of ***S*-HPt** and UV–*vis* absorption spectra of ligands ***R*/*S*-7**

4. Computational investigations

5. Photophysical properties

6. Thermal properties of ***R*/*S*-HPt**.

7. Chiroptical spectra of ***R*/*S*-HPt**

8. SEM images of complexes ***R*/*S*-HPt**

9. Molecular dynamics (MD) simulations data of ***S*-HPt-C4**

10. Cyclic voltammetry (CV) curves of complexes ***R*/*S*-HPt**

11. EL performance of homoleptic complex-based devices

12. MALDI-TOF MS spectra of homoleptic Pt(II) complexes ***R*/*S*-HPt**

13. NMR spectra of homoleptic Pt(II) complexes ***R*/*S*-HPt**

14. HPLC spectra of homoleptic Pt(II) complexes ***R*/*S*-HPt**

15. References

1. Experimental section

Materials and methods. All precursor reagents, solvents and high purity nuclear magnetic resonance (NMR) solvents were purchased from commercial sources (Sigma Aldrich, Adamas, TCI and Acros) and used as supplied unless otherwise indicated. Solvents were distilled from sodium/benzophenone (toluene, tetrahydrofuran, Et_2O, *n*-hexane) or calcium hydride (DCM, $CHCl_3$) under argon prior to use. Chiral N^N-cyclometalated ligands ***R*/*S*-7** (**Scheme S1**) were prepared via the serial reactions of Sonogashira,[1] Suzuki-Miyaura carbon–carbon coupling,[2] and the Sharpless copper-catalyzed Huisgen's 1,3-dipolar cycloaddition procedures.[3] All oxygen or moisture sensitive reactions were performed under argon atmosphere using standard Schlenk method. ^{1}H NMR and ^{13}C NMR spectra were detected on Bruker avance III 400/500 MHz spectrometer. Chemical shifts were recorded as parts per million (ppm, δ) relative to tetramethylsilane (δ 0.00) and $CDCl_3$ (δ = 7.26, singlet). ^{1}H NMR splitting patterns are designated as singlet (s), doublet (d), triplet (t), multiplets (m) and etc. Mass spectrometry (MS) analyses were recorded on a Bruker Daltonics Ultrafle Xtreme MALDI-TOF MS, and α-cyano-4-hydroxycinnamic acid (α-CHCA) was used as supporting matrix in the MALDI-TOF MS measurement. Chiral high-performance liquid chromatography (HPLC) analyses were performed on Shimadzu SPD-20A using Daicel Chiralpak IA Column. Thermo-gravimetry analysis (TGA) was performed on a NETZSCH STA 449 ***F3*** Jupiter instrument in an atmosphere of N_2 at a heating rate of 10 ºC/min. The liquid crystalline textures were investigated and photographed using liquid crystal cells with a polarized optical microscope (POM) equipped with a Leitz-350 heating stage and an associated Nikon (D3100) digital camera. Differential scanning calorimetry (DSC) experiments were performed on a METTLER DSC 823e differential canning calorimeter at a scan rate of 10 ºC/min under the nitrogen atmosphere. Temperature-dependent, powder X-ray diffraction was measured on a small & wide angle X-Ray scattering system (SAXSess mc^2, Cu-Kα, λ = 0.15418 nm). Ultraviolet−visible (UV−*vis*) absorption spectra were measured at room temperature on a Shimadzu UV-3600 spectrophotometer. The photoluminescence (PL) emission spectra were measured on an Edinburgh FLS 980 instrument or a HITACHI F-7000 fluorescence spectrophotometer at room temperature. Absolute quantum yield measured using the calibrated integrating sphere system at room temperature. The time-resolved measurements were taken on an Edinburgh FLS 980 instrument to measure the excited state lifetime. A diode laser with λ = 365 nm was used as the excitation source, and the time-correlated single photo count (TCSPC) method was used to collect photos. Scanning electron microscope (SEM) images were taken in Hitachi S-4800 field emission scanning electron microscopy.

Thermal Annealing Treatments. The thin film was prepared by dissolving the corresponding sample in chloroform (4 mg mL^{-1}), followed by a spin coating (3000 rpm, 30 s) onto the quartz plate. The above quartz plates were put on the hot stage at 140 ºC and the films were kept at this temperature for 30 min. Finally, the quartz plates

were moved to room temperature.

Device Fabrication and Characterization. The solution-processed multilayer OLEDs were fabricated as follows: Indium tin oxide (ITO)-coated glass substrates (1.5 cm × 1.5 cm) were cleaned with deionized water, and then sequentially ultrasonicated in dichloromethane (DCM), acetone and ethanol for 20 min, respectively. After finishing the preparatory work, the ITO substrates were treated in a UV-ozone oven for 20 min. Next, the 25 nm thickness PEDOT:PSS was spin-coated on the ITO substrates as the hole injection layer and dried at 120 ºC for 30 min to remove the solvent. After that, the emitting layer (50 nm) was spin-coated onto the PEDOT:PSS coated substrates and dried at 140 ºC for 30 min under N_2 atmosphere. Subsequently, 1,3,5-tris(N-phenylbenz-imidazol-2-yl)-benzene (TPBi, 35 nm) was deposited by vacuum deposition as the electron transporting layer and hole blocking layer. Finally, Ca (10 nm) and Ag (100 nm) were sequentially deposited under 5×10^{-5} Pa pressure as the electron injection layer and cathode, respectively. The current-voltage, device brightness and electroluminescence spectra characteristics of OLEDs were detected by using the Keithley 2636A and Photo Research PR-655 Spectra Scan.

Electrochemical tests. CV measurements were performed on a CHI660D (Shanghai CH Instrument Company, China). Cyclic voltammograms of ***R*/*S*-HPt** complexes were measured in degassed DCM solution (1.0×10^{-3} mol/L) with 0.1 mol/L tetrabutylammonium hexafluorophosphate (Bu_4NPF_6 was purified by recrystallization in EtOH before being used) as the supporting electrolyte with a scan rate of 100 mV/s. Potentials are reported *vs* the Fc/Fc^+ redox couple as an external reference. Counter electrode: Pt wire, working electrode: glassy carbon, and reference electrode: Ag/AgCl, with KCl (3 mol/L in deionized water) as a filling solution.

Computational details. Computational studies were carried out using Gaussian 16 program package.[4] For density functional theory (DFT) and time-dependent DFT (TDDFT) calculations, B3LYP[5] hybrid density function and 6-311g(d) basis was used for nonmetal atoms along with SMD solvent model (solvent: chloroform).[6] For platinum, a Stuttgart−Dresden (SDD) effective core potential was employed. S1 to S60 excited states were calculated based on the optimized structures in their ground state from DFT results, and the UV−*vis* spectrum was simulated using Multiwfn software.[7] The empirical dispersion correction D3 with Becke-Johnson damping (GD3BJ) was used for all calculations.[8-12]

MD Stimulation Method. All molecular dynamics simulations were carried out using Gromacs 2018.8 program[13] with general Amber ff99SB force field.[14] In this work, all molecules were optimized by Gaussian 16 program (DFT section).[4] Multiwfn was used to construct RESP charges.[7] 21 ***S*-HPt-C4** molecules were put into a cubic box with a side length of 5 nm using packmol software.[15] The cut-off for neighbor list of

Verlet method and that for short-range interactions is 1.2 nm in all calculations with periodic boundary conditions in all three directions. After the energy minimization, the systems were equilibrated in NPT ensemble. In this simulation, the system was simulated for 200 ps using Berendsen thermostat at 413.15 K with a coupling constant of 0.1 ps.[16] The time step of each simulation was taken as 2 fs. Then, all systems were finally run in NVT ensemble for 100 ns at 413.15 K. The time step of each simulation was 2 fs. Complexation energies were corrected for basis set superposition error (BSSE) using the counterpoise correction method.[17]

2. Synthetic procedures and characterization

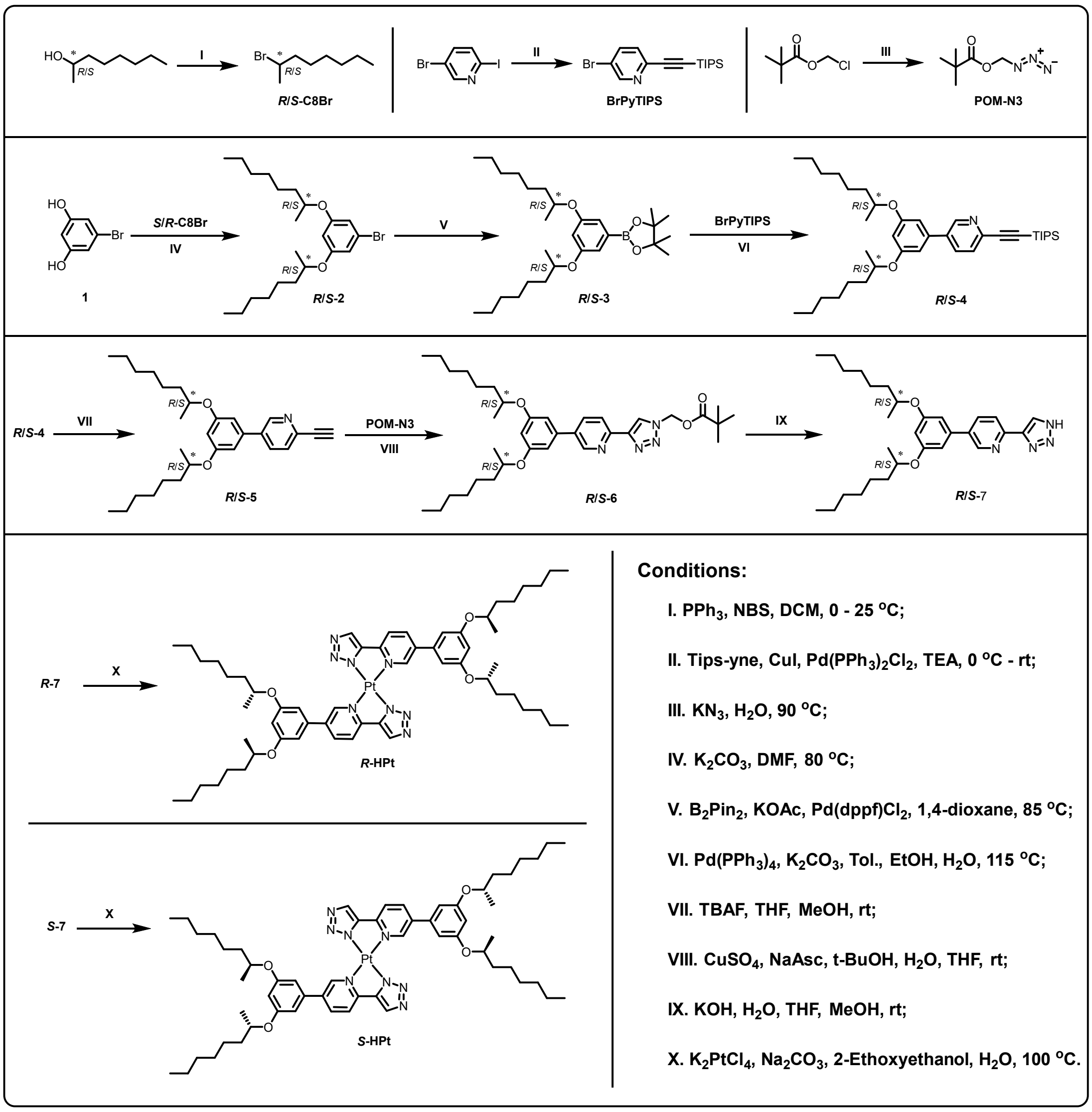

Scheme S1. Synthetic route of homoleptic Pt(II) complexes ***R*/*S*-HPt**.

Synthesis of compound *S*-C8Br[18]

To a mixture of (*S*)-octan-2-ol (10.0 g, 76.79 mmol) and PPh_3 (30.21 g, 115.18 mmol) in dichloromethane (DCM, 60 mL), and N-Bromosuccinimide (NBS, 17.77 g, 99.82 mmol) was added slowly at 0 ºC. Then protect it from light and stir it at room temperature for 24 h. Then, the solution was poured into water (50 mL) and extracted with DCM for three times (3 × 50 mL). The combined organic layers were dried over anhydrous Na_2SO_4 and evaporated to remove solvent in vacuum. The residue was purified by chromatography on silica gel column eluting with PE to yield ***S*-C8Br** as a light brown yellow liquid (12.93 g, 87%). ^{1}H NMR (400 MHz, $CDCl_3$) δ 4.19 – 4.08 (m, 1H), 1.88 – 1.72 (m, 2H), 1.71 (d, *J* = 6.6 Hz, 3H), 1.53 – 1.37 (m, 2H), 1.36 – 1.24 (m, 6H), 0.89 (t, *J* = 6.8 Hz, 3H).

Synthesis of compound BrPyTIPS

To a mixture of 5-bromo-2-iodopyridine (4.72 g, 16.63 mmol), ethynyltriisopropyl-silane (3.06 g, 16.79 mmol), $Pd(PPh_3)_2Cl_2$ (583.46 mg, 831.26 μmol) and CuI (158.31 mg, 831.26 μmol) in triethylamine (TEA, 45 mL) and acetonitrile (MeCN, 45 mL). Under the protection of argon, the mixture was stirred at RT overnight. Then the solvent was removed via evaporating and the residue dissolved in DCM (100 mL), then washed with water (3 × 50 mL), dried over anhydrous Na_2SO_4 and filtrated. The filtrate was evaporated to remove the solvent and the residue was passed through a flash silica gel column using *n*-hexane as the eluent to give **BrPyTIPS** as a clear colorless oil (4.69 g, 83%). ^{1}H NMR (400 MHz, $CDCl_3$) δ 8.64 (d, *J* = 1.9 Hz, 1H), 7.77 (dd, *J* = 8.3, 2.4 Hz, 1H), 7.35 (dd, *J* = 8.3, 0.4 Hz, 1H), 1.16 – 1.12 (m, 21H).

Synthesis of compound POM-N3

To a solution of KN_3 (4.76 g, 58.72 mmol) in deionized water (100 mL) was added chloromethyl pivalate (8.04 g, 53.39 mmol). The mixture was stirred at 90 ºC overnight under the inert gas atmosphere. The resultant mixture was extracted with ethyl acetate (EA, 100 mL) for three times. The final combined organic layer was dried with anhydrous Na_2SO_4 and filtered. The solvent was removed by evaporating to get **POM-N3** as a colorless liquid (8.00 g, 95%). ^{1}H NMR (400 MHz, $CDCl_3$) δ 5.14 (s, 2H), 1.25 (s, 9H). The NMR matched the reported previously.[19]

Synthesis of compound *S*-2[20-23]

To a mixture of 5-bromobenzene-1,3-diol (5.75 g, 30.42 mmol), ***S*-C8Br** (12.93 g, 66.93 mmol) and potassium carbonate (16.82 g, 121.69 mmol) was added a degassed mixture of N, N-Dimethylformamide (DMF, 50 mL). The mixture was reacted at 80 ºC

for 36 h under the protection of argon. After cooled to RT, the mixture was poured into water (100 mL) and extracted with DCM (3 × 50 mL). The combined organic layer was dried over anhydrous Na_2SO_4 and filtered. The filtrate was evaporated to remove the solvent and the residue was passed through a flash silica gel column using PE/DCM (10:1) as the eluent to get ***S*-2** as a colorless oil (10.00 g, 79%).^{1}H NMR (400 MHz, $CDCl_3$) δ 6.60 (d, J = 2.2 Hz, 2H), 6.33 (s, 1H), 4.32 – 4.23 (m, 2H), 1.69 (ddd, J = 11.5, 9.5, 5.6 Hz, 2H), 1.53 (ddd, J = 13.5, 10.4, 5.1 Hz, 2H), 1.45 – 1.38 (m, 2H), 1.29 (dd, J = 14.2, 3.7 Hz, 20H), 0.88 (t, J = 6.8 Hz, 6H).

Synthesis of compound *S*-3

A mixture of ***S*-2** (3.50 g, 8.47 mmol), bis(pinacolato)diboron (2.58 g, 10.16 mmol), potassium acetate (2.49 g, 25.40 mmol), [1,1'-bis(dipheny- lphosphino)-ferrocene] dichloropalla- dium complex with dichloromethane (1:1) (691.33 mg, 846.56 μmol), and 1,4-dioxane (60 mL) was stirred at 85 ºC for 12 h under argon atmosphere. The resulting mixture was cooled to RT, poured into ice-water (100 mL) and then extracted with DCM (3 × 50 mL). The combined organic layers were washed with water and dried over anhydrous Na_2SO_4 and filtrated. The filtrate was evaporated to remove the solvent and the residue was passed through a flash silica gel column using PE/EA (10:1) as the eluent to give ***S*-3** as a brown oil (3.41 g, 88%). ^{1}H NMR (400 MHz, $CDCl_3$) δ 6.91 (d, J = 2.3 Hz, 2H), 6.53 (t, J = 2.3 Hz, 1H), 4.38 (dd, J = 12.1, 6.1 Hz, 2H), 1.71 (ddd, J = 13.2, 9.7, 6.6 Hz, 2H), 1.59 – 1.51 (m, 2H), 1.47 – 1.40 (m, 2H), 1.33 (s, 12H), 1.29 (dd, J = 10.7, 5.8 Hz, 20H), 0.88 (t, J = 6.8 Hz, 6H).

Synthesis of compound *S*-4

To a mixture of ***S*-3** (3.41 g, 7.40 mmol), **BrPyTIPS** (2.76 g, 8.15 mmol), potassium carbonate (3.07 g, 22.21 mmol) and tetrakis(triphenylphosphine) palladium ($Pd(PPh_3)_4$, 855.70 mg, 740.49 μmol) was added a degassed mixture of toluene (40 mL), water (20 mL) and ethanol (20 mL). The mixture was refluxed (115 ºC) for 24 h under the protection of argon. After cooled to RT, the mixture was poured into water (150 mL) and extracted with DCM (3 × 50mL). The combined organic layer was dried over anhydrous Na_2SO_4 and filtered. The filtrate was evaporated to remove the solvent and the residue was passed through a flash silica gel column using PE/EA (30:1) as the eluent to give ***S*-4** as a brown oil (4.30 g, 98%). ^{1}H NMR (400 MHz, $CDCl_3$) δ 8.79 (d, J = 1.5 Hz, 1H), 7.85 (d, J = 7.8 Hz, 1H), 7.54 (d, J = 8.1 Hz, 1H), 6.63 (d, J = 2.0 Hz, 2H), 6.48 (d, J = 1.9 Hz, 1H), 4.43 – 4.33 (m, 2H), 1.79 – 1.70 (m, 2H), 1.58 (ddd, J = 13.6, 10.5, 5.1 Hz, 2H), 1.45 (dd, J = 11.5, 6.0 Hz, 2H), 1.39 – 1.25 (m, 20H), 1.22 – 1.10 (m, 21H), 0.88 (t, J = 6.9 Hz, 6H).

Synthesis of compound *S*-5

To a solution of ***S*-4** (4.30 g, 7.26 mmol) and tetrabutylammonium fluoride (TBAF, 2.85 g, 10.90 mmol) in tetrahydrofuran (THF, 20 mL) and methanol (MeOH; 2 mL). The mixture was stirred at RT for 5 min under the air atmosphere. After reaction completion (detected by thin layer chromatography, TLC), the mixture was evaporated to remove the solvent and the residue was passed through a flash silica gel column using PE/EA (10:1) as the eluent to give ***S*-5** as a colorless oil (3.01 g, 95%). ^{1}H NMR (400 MHz, $CDCl_3$) δ 8.80 (d, J = 1.7 Hz, 1H), 7.85 (dd, J = 8.1, 2.3 Hz, 1H), 7.54 (d, J = 8.1 Hz, 1H), 6.65 (d, J = 2.1 Hz, 2H), 6.48 (t, J = 2.1 Hz, 1H), 4.38 (dt, J = 12.1, 6.1 Hz, 2H), 3.24 (s, 1H), 1.80 – 1.71 (m, 2H), 1.58 (ddd, J = 13.6, 10.6, 5.1 Hz, 2H), 1.48 – 1.41 (m, 2H), 1.38 – 1.24 (m, 20H), 0.88 (t, J = 6.9 Hz, 6H).

Synthesis of compound *S*-6

A solution of ***S*-5** (3.61 g, 8.29 mmol) and **POM-N3** (1.56 g, 9.94 mmol) in THF (30 mL), was added copper sulfate pentahydrate (206.89 mg, 828.64 μmol) and sodium ascorbate (541.72 mg, 2.73 mmol) in deionized water (10 mL) and tert-butanol (5 mL). The mixture was stirred overnight at RT in air. After reaction completion (detected by TLC), the mixture was extracted with EA (3 × 20 mL). The combined organic layer was washed with water (3 × 50 mL), and dried over anhydrous Na_2SO_4 and filtrated. The filtrate was evaporated to remove the solvent and the residue was passed through a flash silica gel column using PE/EA (5:1) as the eluent to give ***S*-6** as a light brown oil (3.08 g, 63 %). ^{1}H NMR (400 MHz, $CDCl_3$) δ 8.82 (s, 1H), 8.31 (s, 1H), 8.05 (s, 1H), 6.69 (d, J = 2.1 Hz, 2H), 6.49 (s, 1H), 6.33 (s, 2H), 4.40 (dd, J = 12.1, 6.1 Hz, 2H), 1.81 – 1.71 (m, 2H), 1.64 – 1.54 (m, 2H), 1.46 (dd, J = 11.6, 6.0 Hz, 2H), 1.42 – 1.26 (m, 21H), 1.21 (s, 9H), 0.88 (t, J = 6.8 Hz, 6H).

Synthesis of compound *S*-7[3]

A solution of ***S*-6** (4.91 g, 8.28 mmol) in THF (30 mL), was added KOH (1.02 g, 18.22 mmol; 2 M in water) and MeOH (5 mL). The mixture was stirred at RT in air for 30 min, after reaction completion (detected by TLC), the mixture was extracted with EA (3 × 20 mL). The combined organic layer was washed with water (3 × 50 mL), and dried over anhydrous Na_2SO_4 and filtrated. The filtrate was evaporated to remove the solvent to give crude product ***S*-7** as a colorless oil (2.54 g, 64%). This product was directly used to next step without further purification.

Synthesis of chiral Pt(II) complex *S*-HPt[19]

To a mixture of ***S*-7** (500.00 mg, 1.04 mmol), K_2PtCl_4 (197.08 mg, 474.79 μmol) and Na_2CO_3 (251.61 mg, 2.37 mmol) in 2-Ethoxyethanol (10 mL) and water (3.5 mL), was stirred at 100 ºC for 24 h. After the mixture was cooled to RT, it was extracted with DCM (3 × 10 mL). The combined organic layer was washed with water (3 × 20 mL), and dried over anhydrous Na_2SO_4 and filtrated. The filtrate was evaporated to remove the solvent and the residue was passed through a flash silica gel column using DCM/EA (10:1) as the eluent to give ***S*-HPt** as an orange-red waxy solid (500 mg, 88%). ^{1}H NMR (500 MHz, $CDCl_3$) δ 10.56 (s, 2H), 8.09 – 8.04 (m, 2H), 7.85 (s, 2H), 7.56 (dd, *J* = 8.1, 3.5 Hz, 2H), 6.82 (s, 4H), 6.54 (s, 2H), 4.52 (dd, *J* = 11.9, 5.9 Hz, 4H), 1.81 (ddd, *J* = 16.5, 10.8, 5.9 Hz, 4H), 1.66 (ddd, *J* = 13.7, 10.6, 5.3 Hz, 4H), 1.53 (dt, *J* = 17.2, 6.1 Hz, 4H), 1.44 (d, J = 6.0 Hz, 16H), 1.39 – 1.35 (m, 8H), 1.33 – 1.29 (m, 16H), 0.89 (t, *J* = 6.6 Hz, 12H). ^{13}C NMR (126 MHz, $CDCl_3$) δ 160.20, 151.60, 150.07, 144.47, 137.39, 136.64, 136.08, 131.02, 119.02, 106.44, 104.43, 74.26, 36.65, 31.87, 29.39, 25.57, 22.66, 19.86, 14.13. MALDI-TOF MS m/z (α-CHCA as matrix) calcd. for $C_{58}H_{83}N_8O_4Pt^+$ $[M+H]^+$: m/z = 1150.619, found: m/z = 1150.083.

Synthesis of chiral Pt(II) complex *R*-HPt. The chiral Pt(II) complex ***R*-HPt** (yield = 93%) and its organic precursors were synthesized according to the same methods of complex ***S*-HPt**. ^{1}H NMR (500 MHz, $CDCl_3$) δ 10.61 (s, 2H), 8.12 – 8.04 (m, 2H), 7.89 (s, 2H), 7.59 (d, *J* = 8.2 Hz, 2H), 6.83 (d, *J* = 1.2 Hz, 4H), 6.54 (t, *J* = 1.8 Hz, 2H), 4.53 (dt, *J* = 12.0, 5.9 Hz, 4H), 1.81 (dq, *J* = 13.4, 5.7 Hz, 4H), 1.71 – 1.61 (m, 4H), 1.56 – 1.49 (m, 4H), 1.43 (d, *J* = 6.0 Hz, 16H), 1.39 – 1.34 (m, 8H), 1.33 – 1.29 (m, 16H), 0.88 (t, *J* = 6.6 Hz, 12H). ^{13}C NMR (126 MHz, $CDCl_3$) δ 160.19, 151.59, 150.08, 144.45, 137.36, 136.65, 136.02, 131.05, 119.01, 106.45, 104.39, 74.26, 36.65, 31.87, 29.39, 25.58, 22.66, 19.86, 14.13. MALDI-TOF MS m/z (α-CHCA as matrix) calcd. for $C_{58}H_{83}N_8O_4Pt^+$ $[M+H]^+$: m/z = 1150.619, found: m/z = 1150.059.

3. UV–*vis* absorption and PL spectra of *S*-HPt and UV–*vis* absorption spectra of ligands *R*/*S*-7

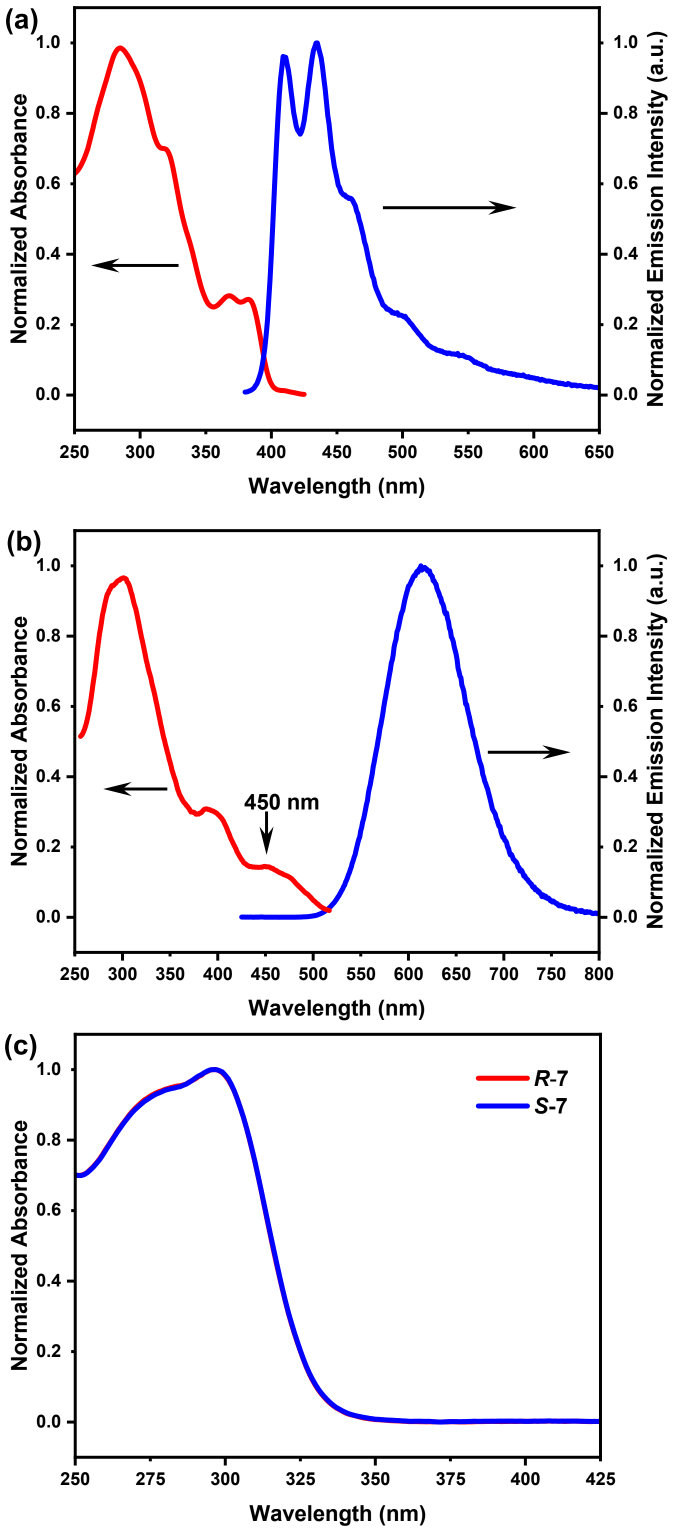

Fig. S1 UV–*vis* absorption and PL spectra of ***S*-HPt** in chloroform (1.0 × 10^{-5} mol L^{-1}) (a) and spin-coated films (b); UV–*vis* absorption spectra of ligands ***R*/*S*-7** in chloroform (1.0 × 10^{-5} mol L^{-1}) (c).

4. Computational investigations

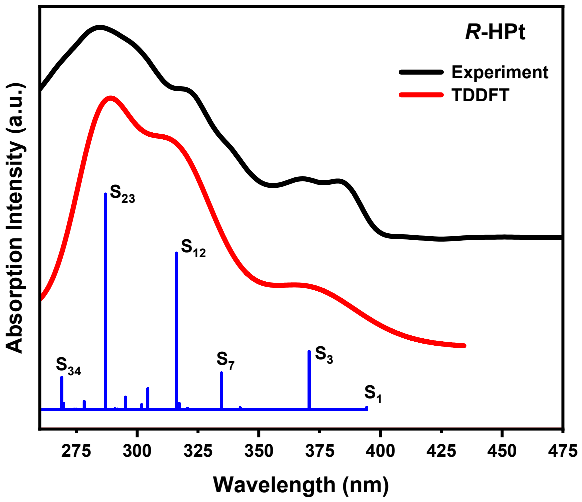

Fig. S2 TDDFT-predicated vertical excitations of ***R*-HPt**. The absorption spectrum is shown for comparison.

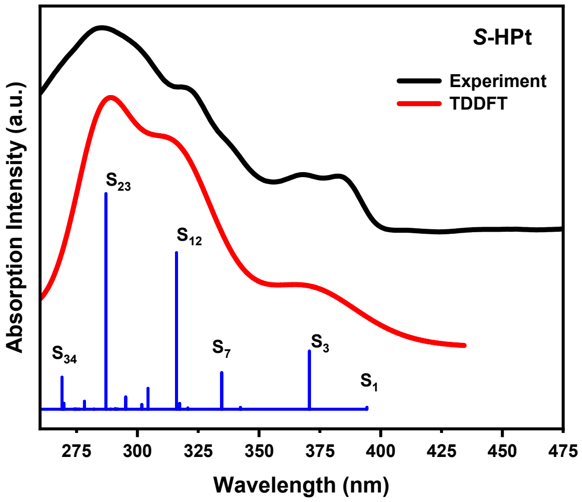

Fig. S3 TDDFT-predicated vertical excitations of ***S*-HPt**. The absorption spectrum is shown for comparison.

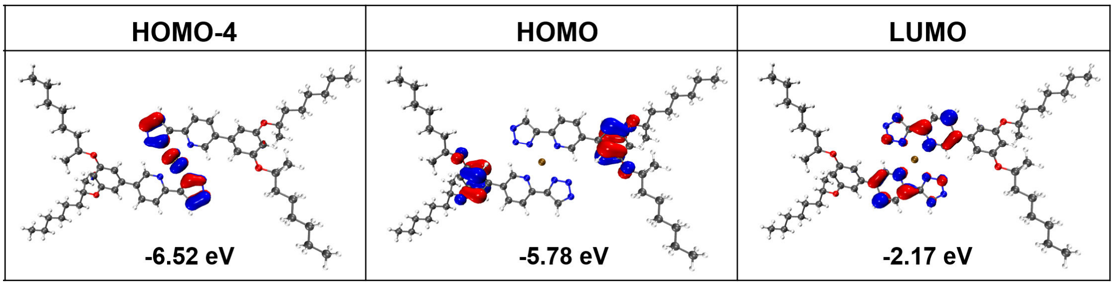

Fig. S4 Frontier molecular orbitals and energy level of ***S*-HPt**.

Table S1 Computed excitation energies and oscillator strengths for the $S_1 \rightarrow S_{60}$ transitions of homoleptic Pt(II) complexes ***R*-HPt**.

S_n	*E* (eV)	λ (nm)	*f*	transitions
S_1	3.1444	394.3	0.0095	H → L 93.9%, H-1 → L+1 5.1%
S_2	3.1445	394.29	0.0029	H-1 → L 93.9%, H → L+1 5.2%
S_3	3.3445	370.71	0.2889	H-2 → L 78.8%, H-4 → L 18.2%
S_4	3.4683	357.48	0	H-3 → L 96.0%
S_5	3.6214	342.37	0.0054	H → L+1 93.4%, H-1 → L 5.1%
S_6	3.6216	342.35	0.0089	H-1 → L+1 93.0%, H → L 5.1%
S_7	3.705	334.64	0.1819	H-4 → L 78.1%, H-2 → L 18.8%
S_8	3.8271	323.96	0	H-2 → L+1 83.5%, H-4 → L+1 9.8%
S_9	3.8657	320.73	0.006	H-7 → L 98.5%
S_{10}	3.9066	317.37	0.029	H → L+2 55.2%, H-1 → L+3 33.0%, H-3 → L+1 8.0%
S_{11}	3.909	317.18	0.0086	H-1 → L+2 60.6%, H → L+3 36.0%
S_{12}	3.9226	316.08	0.777	H-3 → L+1 80.6%, H → L+2 5.8%, H-5 → L 5.6%
S_{13}	4.0737	304.35	0.1031	H-5 → L 64.3%, H-8 → L 22.8%, H-3 → L+1 7.4%
S_{14}	4.0952	302.75	0.0001	H-4 → L+1 49.7%, H-6 → L 33.6%, H-2 → L+1 8.3%
S_{15}	4.1079	301.82	0.024	H-4 → L+4 53.0%, H-2 → L+4 26.9%, H-2 → L+2 7.7%
S_{16}	4.1579	298.19	0.0001	H-6 → L 55.4%, H-4 → L+1 34.0%
S_{17}	4.2003	295.18	0.061	H-2 → L+2 38.8%, H-8 → L 25.6%, H-3 → L+3 12.2%, H-5 → L 10.4%
S_{18}	4.2484	291.84	0	H → L+3 62.2%, H-1 → L+2 37.0%
S_{19}	4.2485	291.83	0.0016	H-1 → L+3 62.0%, H → L+2 36.7%
S_{20}	4.2626	290.87	0.0032	H-7 → L+1 92.8%
S_{21}	4.271	290.29	0.0002	H-7 → L+4 38.5%, H-3 → L+2 25.2%, H-2 → L+3 21.5%, H-8 → L+1 7.7%
S_{22}	4.29	289.01	0.0003	H-7 → L+4 45.9%, H-2 → L+3 21.3%, H-3 → L+2 18.7%, H-6 → L 5.7%
S_{23}	4.319	287.07	1.0709	H-8 → L 44.7%, H-2 → L+2 31.0%, H-5 → L 13.8%
S_{24}	4.3943	282.15	0.0004	H-9 → L 93.9%
S_{25}	4.4255	280.16	0	H-3 → L+2 46.7%, H-2 → L+3 42.1%, H-4 → L+3 5.6%
S_{26}	4.4562	278.23	0.04	H-3 → L+3 71.0%, H-2 → L+2 13.6%, H-4 → L+2 9.8%
S_{27}	4.4915	276.04	0.0008	H-10 → L 53.3%, H-2 → L+4 14.2%, H-8 → L+4 7.5%, H-4 → L+4 6.7%, H-4 → L+2 5.7%
S_{28}	4.51	274.91	0.002	H-10 → L 35.2%, H-2 → L+4 16.5%, H-8 → L+4 12.6%, H-4 → L+4 9.8%, H-4 → L+2 9.7%, H-5 → L+4 6.2%
S_{29}	4.5226	274.14	0.0001	H-1 → L+4 96.4%
S_{30}	4.524	274.06	0.0018	H → L+4 96.4%
S_{31}	4.5688	271.37	0	H-5 → L+1 89.6%
S_{32}	4.5922	269.99	0	H-3 → L+4 81.8%, H-4 → L+3 6.5%, H-6 → L+4 5.7%
S_{33}	4.5963	269.75	0.0296	H-6 → L+1 41.4%, H-4 → L+2 36.0%, H-3 → L+3 7.4%, H-4 → L+4 5.4%
S_{34}	4.6089	269.01	0.1591	H-6 → L+1 50.7%, H-4 → L+2 32.9%
S_{35}	4.6331	267.61	0	H-4 → L+3 82.5%, H-3 → L+4 5.1%
S_{36}	4.7591	260.52	0.0002	H-8 → L+1 72.8%, H-2 → L+3 6.3%

S_{37}	4.8006	258.27	0.0031	H-8 → L+4 51.5%, H-2 → L+4 28.5%
S_{38}	4.8275	256.83	0	H-7 → L+2 94.0%
S_{39}	4.8659	254.8	0.0006	H-7 → L+3 90.9%
S_{40}	4.9058	252.73	0.0121	H-9 → L+1 76.8%, H-10 → L 7.6%, H-5 → L+2 7.2%
S_{41}	4.9163	252.19	0.0835	H-5 → L+2 56.7%, H-6 → L+3 22.5%, H-9 → L+1 9.1%
S_{42}	4.9294	251.52	0.0001	H-6 → L+2 45.8%, H-5 → L+3 24.1%, H-11 → L 18.0%
S_{43}	4.9982	248.06	0	H-10 → L+1 91.1%
S_{44}	5.0213	246.92	0.0038	H-11 → L 55.1%, H-1 → L+5 11.3%, H-13 → L 7.0%, H-5 → L+3 6.8%, H → L+6 5.8%
S_{45}	5.0441	245.8	0.0349	H → L+5 48.8%, H-1 → L+6 25.1%, H-3 → L+8 7.3%
S_{46}	5.0503	245.5	0.0141	H-1 → L+5 37.7%, H → L+6 19.4%, H-11 → L 11.4%, H-2 → L+8 6.0%
S_{47}	5.1308	241.65	0.0343	H-8 → L+2 28.8%, H-6 → L+3 27.9%, H-12 → L 23.2%, H-5 → L+2 11.1%
S_{48}	5.1403	241.2	0.0001	H-5 → L+3 51.8%, H-6 → L+2 35.6%
S_{49}	5.1758	239.55	0.0261	H-6 → L+3 41.5%, H-12 → L 22.3%, H-8 → L+2 15.9%, H-5 → L+2 14.1%
S_{50}	5.1778	239.45	0	H-19 → L+4 44.6%, H-9 → L+4 43.6%, H-9 → L+2 5.1%
S_{51}	5.2476	236.27	0.1195	H-12 → L 45.5%, H-8 → L+2 42.8%
S_{52}	5.2824	234.71	0	H-8 → L+3 48.4%, H-13 → L 36.0%
S_{53}	5.3219	232.97	0	H-13 → L 42.5%, H-8 → L+3 37.0%, H-11 → L 7.3%
S_{54}	5.3556	231.5	0.0017	H-16 → L 90.6%
S_{55}	5.3828	230.33	0	H-6 → L+4 58.2%, H-9 → L+2 25.8%, H-19 → L 6.3%
S_{56}	5.3839	230.29	0.0022	H-5 → L+4 78.9%, H-8 → L+4 7.5%
S_{57}	5.392	229.94	0	H-9 → L+2 49.2%, H-6 → L+4 30.1%, H-19 → L 9.4%
S_{58}	5.4221	228.66	0.0002	H-19 → L 77.6%, H-9 → L+2 16.2%
S_{59}	5.4442	227.74	0.0626	H-11 → L+1 53.7%, H-2 → L+5 12.6%, H → L+5 6.7%, H → L+7 5.5%
S_{60}	5.4549	227.29	0.0009	H-11 → L+1 30.5%, H → L+5 20.6%, H-1 → L+6 16.4%, H-2 → L+5 12.7%

Table S2 Computed excitation energies and oscillator strengths for the $S_1 \rightarrow S_{60}$ transitions of homoleptic Pt(II) complexes ***S*-HPt**.

S_n	E (eV)	λ (nm)	f	transitions
S_1	3.1444	394.3	0.0095	H → L 93.9%, H-1 → L+1 5.1%
S_2	3.1445	394.29	0.0029	H-1 → L 93.9%, H → L+1 5.2%
S_3	3.3445	370.71	0.2889	H-2 → L 78.8%, H-4 → L 18.2%
S_4	3.4683	357.48	0	H-3 → L 96.0%
S_5	3.6214	342.37	0.0054	H → L+1 93.3%, H-1 → L 5.1%
S_6	3.6216	342.35	0.0089	H-1 → L+1 93.0%, H → L 5.1%
S_7	3.705	334.64	0.1819	H-4 → L 78.1%, H-2 → L 18.8%
S_8	3.8271	323.96	0	H-2 → L+1 83.5%, H-4 → L+1 9.8%
S_9	3.8657	320.73	0.006	H-7 → L 98.5%
S_{10}	3.9066	317.37	0.0291	H → L+2 55.2%, H-1 → L+3 32.9%, H-3 → L+1 8.0%
S_{11}	3.909	317.18	0.0086	H-1 → L+2 60.6%, H → L+3 36.0%
S_{12}	3.9225	316.08	0.777	H-3 → L+1 80.6%, H → L+2 5.9%, H-5 → L 5.6%
S_{13}	4.0737	304.35	0.1031	H-5 → L 64.3%, H-8 → L 22.8%, H-3 → L+1 7.4%
S_{14}	4.0952	302.75	0.0001	H-4 → L+1 49.8%, H-6 → L 33.6%, H-2 → L+1 8.3%
S_{15}	4.1079	301.82	0.024	H-4 → L+4 53.0%, H-2 → L+4 26.9%, H-2 → L+2 7.7%
S_{16}	4.1579	298.19	0.0001	H-6 → L 55.4%, H-4 → L+1 33.9%
S_{17}	4.2003	295.18	0.0611	H-2 → L+2 38.8%, H-8 → L 25.6%, H-3 → L+3 12.2%, H-5 → L 10.4%
S_{18}	4.2484	291.84	0	H → L+3 62.2%, H-1 → L+2 36.9%
S_{19}	4.2485	291.83	0.0016	H-1 → L+3 62.0%, H → L+2 36.7%
S_{20}	4.2625	290.87	0.0032	H-7 → L+1 92.8%
S_{21}	4.271	290.29	0.0002	H-7 → L+4 38.5%, H-3 → L+2 25.3%, H-2 → L+3 21.5%, H-8 → L+1 7.7%
S_{22}	4.2899	289.01	0.0003	H-7 → L+4 45.9%, H-2 → L+3 21.3%, H-3 → L+2 18.7%, H-6 → L 5.7%
S_{23}	4.319	287.07	1.0708	H-8 → L 44.7%, H-2 → L+2 31.0%, H-5 → L 13.8%
S_{24}	4.3942	282.15	0.0004	H-9 → L 93.9%
S_{25}	4.4255	280.16	0	H-3 → L+2 46.7%, H-2 → L+3 42.1%, H-4 → L+3 5.6%
S_{26}	4.4562	278.23	0.04	H-3 → L+3 71.0%, H-2 → L+2 13.6%, H-4 → L+2 9.8%
S_{27}	4.4915	276.04	0.0008	H-10 → L 53.4%, H-2 → L+4 14.2%, H-8 → L+4 7.5%, H-4 → L+4 6.7%, H-4 → L+2 5.7%
S_{28}	4.51	274.91	0.002	H-10 → L 35.2%, H-2 → L+4 16.5%, H-8 → L+4 12.6%, H-4 → L+4 9.8%, H-4 → L+2 9.7%, H-5 → L+4 6.2%
S_{29}	4.5226	274.14	0.0001	H-1 → L+4 96.3%
S_{30}	4.524	274.06	0.0018	H → L+4 96.4%
S_{31}	4.5688	271.37	0	H-5 → L+1 89.6%
S_{32}	4.5922	269.99	0	H-3 → L+4 81.8%, H-4 → L+3 6.5%, H-6 → L+4 5.7%
S_{33}	4.5963	269.75	0.0295	H-6 → L+1 41.3%, H-4 → L+2 36.0%, H-3 → L+3 7.4%, H-4 → L+4 5.4%
S_{34}	4.6089	269.01	0.1592	H-6 → L+1 50.7%, H-4 → L+2 32.8%
S_{35}	4.6331	267.61	0	H-4 → L+3 82.5%, H-3 → L+4 5.1%
S_{36}	4.759	260.53	0.0002	H-8 → L+1 72.8%, H-2 → L+3 6.3%

S_{37}	4.8006	258.27	0.0031	H-8 → L+4 51.5%, H-2 → L+4 28.5%
S_{38}	4.8275	256.83	0	H-7 → L+2 94.0%
S_{39}	4.8659	254.8	0.0006	H-7 → L+3 90.9%
S_{40}	4.9058	252.73	0.0121	H-9 → L+1 76.8%, H-10 → L 7.6%, H-5 → L+2 7.2%
S_{41}	4.9163	252.19	0.0835	H-5 → L+2 56.7%, H-6 → L+3 22.5%, H-9 → L+1 9.1%
S_{42}	4.9294	251.52	0.0001	H-6 → L+2 45.8%, H-5 → L+3 24.1%, H-11 → L 18.0%
S_{43}	4.9982	248.06	0	H-10 → L+1 91.1%
S_{44}	5.0213	246.92	0.0038	H-11 → L 55.1%, H-1 → L+5 11.3%, H-13 → L 7.0%, H-5 → L+3 6.8%, H → L+6 5.8%
S_{45}	5.0441	245.8	0.0349	H → L+5 48.8%, H-1 → L+6 25.1%, H-3 → L+8 7.3%
S_{46}	5.0503	245.5	0.0141	H-1 → L+5 37.7%, H → L+6 19.4%, H-11 → L 11.4%, H-2 → L+8 6.0%
S_{47}	5.1308	241.65	0.0343	H-8 → L+2 28.8%, H-6 → L+3 27.9%, H-12 → L 23.2%, H-5 → L+2 11.1%
S_{48}	5.1403	241.2	0.0001	H-5 → L+3 51.8%, H-6 → L+2 35.6%
S_{49}	5.1758	239.55	0.0262	H-6 → L+3 41.5%, H-12 → L 22.3%, H-8 → L+2 15.9%, H-5 → L+2 14.1%
S_{50}	5.1778	239.45	0	H-19 → L+4 44.6%, H-9 → L+4 43.6%, H-9 → L+2 5.1%
S_{51}	5.2476	236.27	0.1195	H-12 → L 45.5%, H-8 → L+2 42.8%
S_{52}	5.2824	234.71	0	H-8 → L+3 48.4%, H-13 → L 36.0%
S_{53}	5.3219	232.97	0	H-13 → L 42.5%, H-8 → L+3 37.0%, H-11 → L 7.3%
S_{54}	5.3556	231.5	0.0017	H-16 → L 90.6%
S_{55}	5.3828	230.33	0	H-6 → L+4 58.1%, H-9 → L+2 26.0%, H-19 → L 6.3%
S_{56}	5.3839	230.29	0.0022	H-5 → L+4 78.9%, H-8 → L+4 7.5%
S_{57}	5.392	229.94	0	H-9 → L+2 49.1%, H-6 → L+4 30.3%, H-19 → L 9.3%
S_{58}	5.4221	228.66	0.0002	H-19 → L 77.6%, H-9 → L+2 16.1%
S_{59}	5.4442	227.74	0.0626	H-11 → L+1 53.7%, H-2 → L+5 12.5%, H → L+5 6.7%, H → L+7 5.5%
S_{60}	5.4548	227.29	0.0009	H-11 → L+1 30.4%, H → L+5 20.6%, H-1 → L+6 16.4%, H-2 → L+5 12.7%

5. Photophysical properties

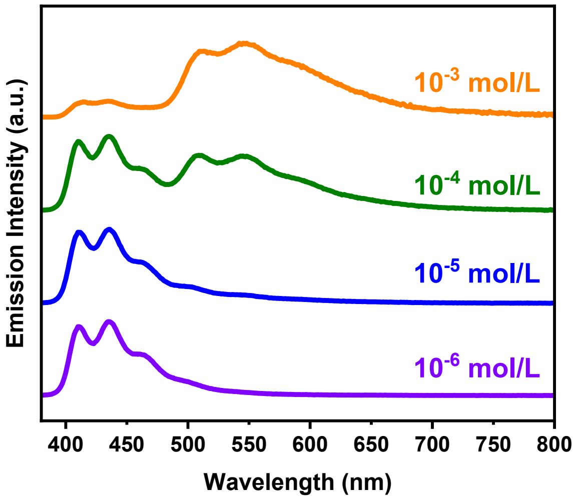

Fig. S5 PL spectra of ***S*-HPt** in chloroform.

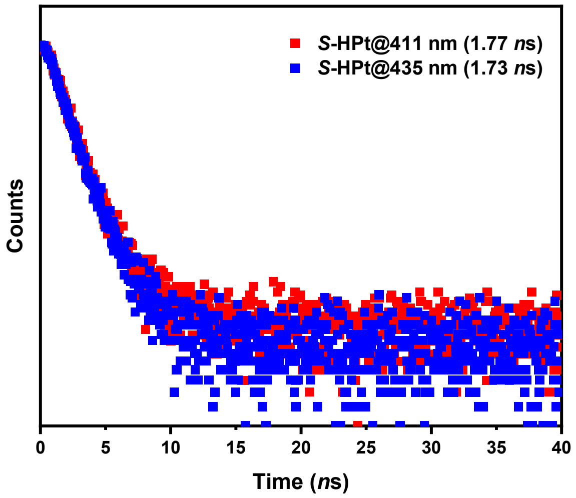

Fig. S6 Transient PL decay of ***S*-HPt** in chloroform (1.0×10^{-6} mol L^{-1}).

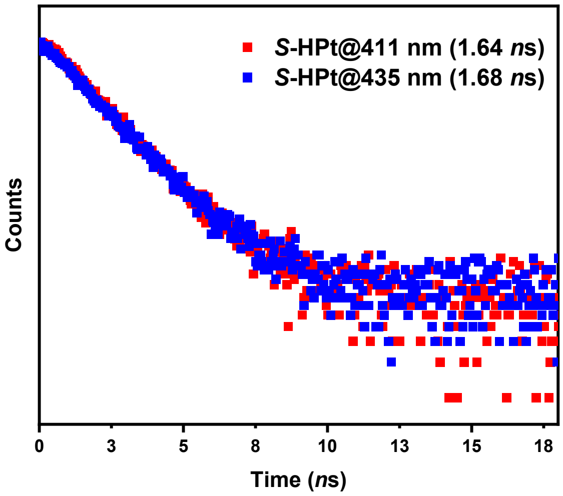

Fig. S7 Transient PL decay of ***S*-HPt** in chloroform (1.0×10^{-5} mol L^{-1}).

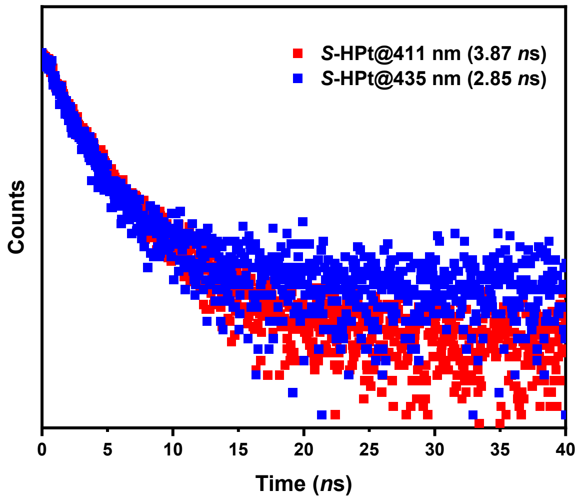

Fig. S8 Transient PL decay of ***S*-HPt** @411 nm and 435 nm in chloroform (1.0×10^{-4} mol L^{-1}).

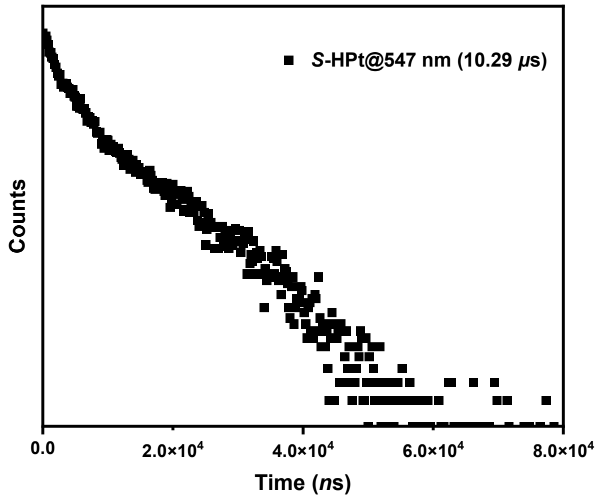

Fig. S9 Transient PL decay of ***S*-HPt** in chloroform (1.0×10^{-3} mol L^{-1}).

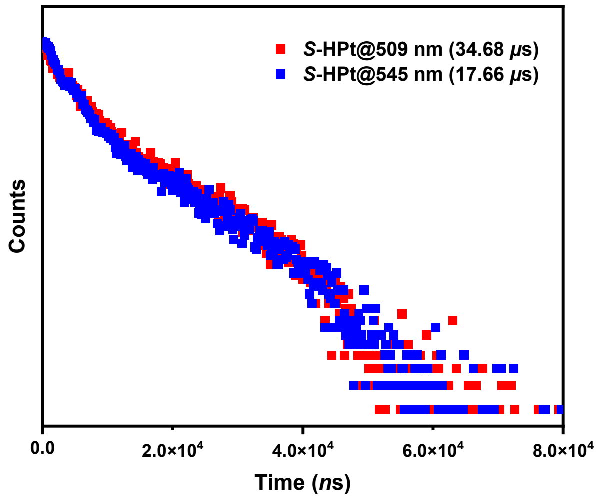

Fig. S10 Transient PL decay of ***S*-HPt** @ 509 nm and 545 nm in chloroform (1.0×10^{-4} mol L^{-1}).

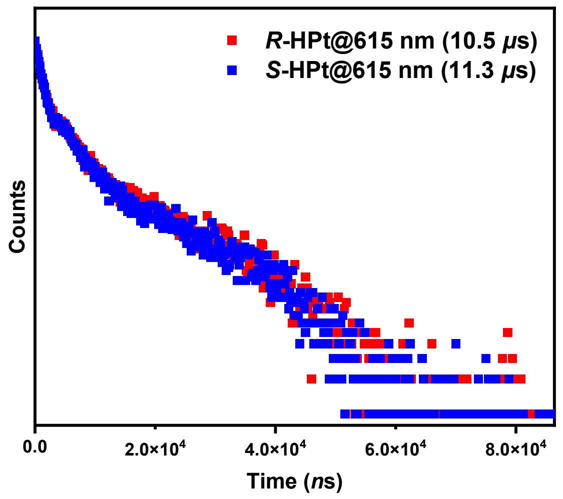

Fig. S11 Transient PL decay of spin-coated films ***R*/*S*-HPt**.

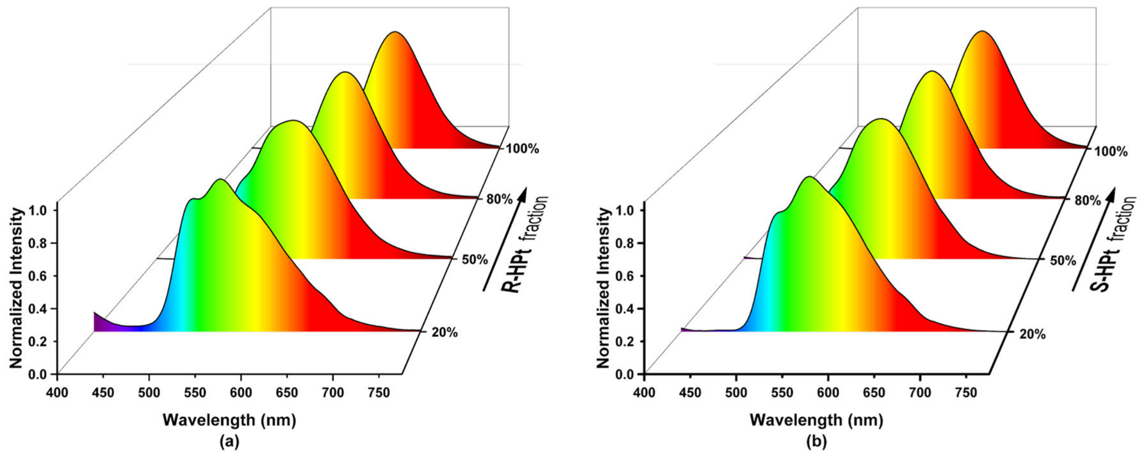

Fig. S12 PL emission spectra of ***R*/*S*-HPt** doped in OXD-7.

6. Thermal properties of *R*/*S*-HPt

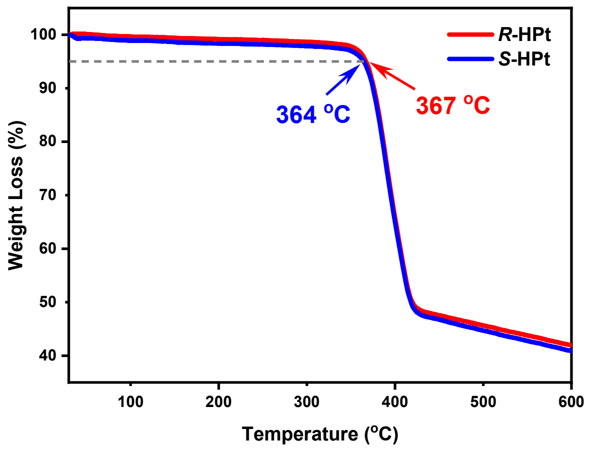

Fig. S13 TGA curves of ***R*/*S*-HPt** under the protection of nitrogen.

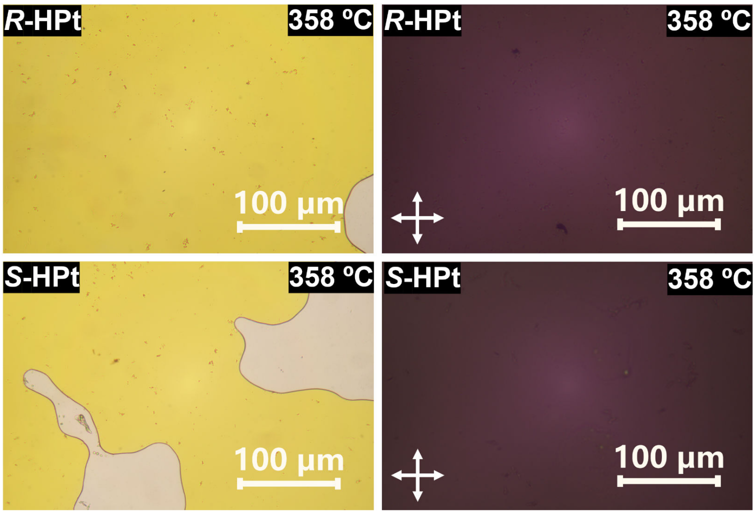

Fig. S14 POM images of ***R*/*S*-HPt** at 358 °C.

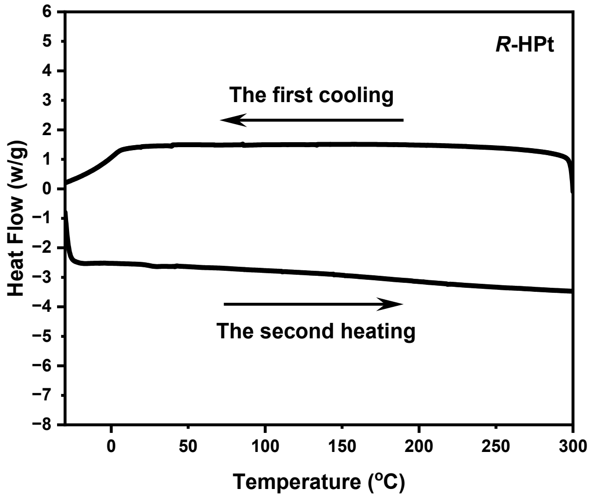

Fig. S15 DSC curves of ***R*-HPt** on the first cooling and the second heating.

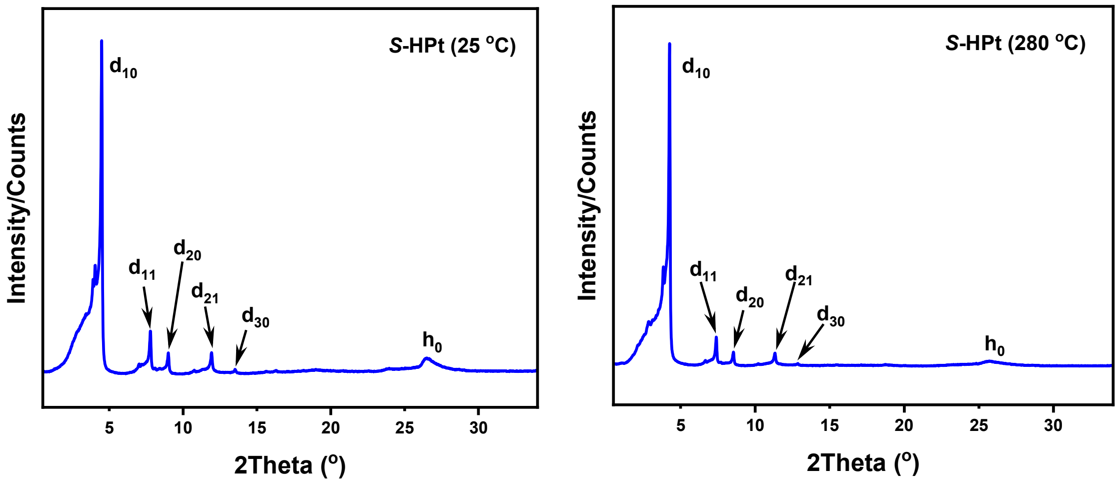

Fig. S16 XRD profiles of ***S*-HPt** at 25 °C and 280 °C on cooling.

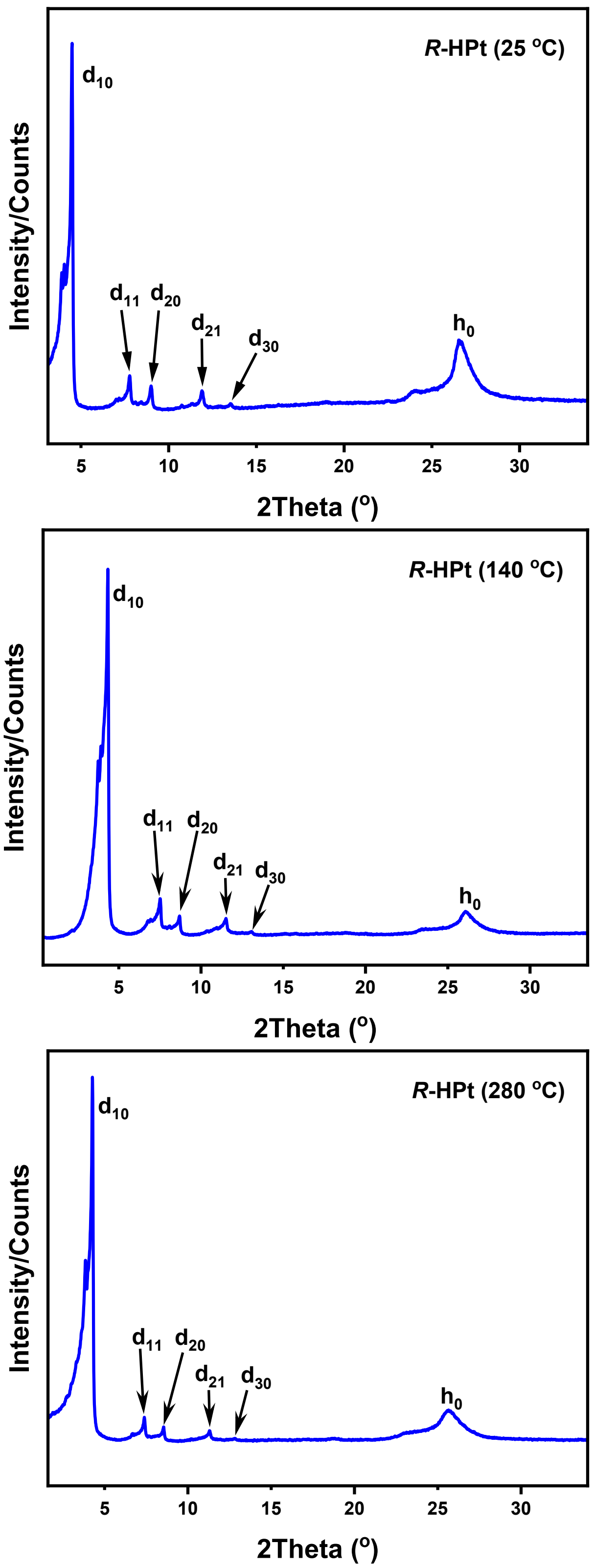

Fig. S17 XRD profiles of ***R*-HPt** at 25 ºC, 140 ºC and 280 ºC on cooling.

Table S3 XRD data of ***R*/*S*-HPt** at different temperature on cooling.

Complexes	*T* (°C)	Phase	q (1/nm)	2θ (°)	d (Å)$_{Mea}$[a]	d (Å)$_{Cal}$	hk[b]	Lattice constants (Å)[c]
***R*-HPt**	25	Col_h	3.19	4.49	19.69	19.69	10	*a* = 22.74
			5.52	7.78	11.38	11.37	11	
			6.38	9.01	9.84	9.85	20	
			8.43	11.89	7.45	7.44	21	
			9.57	13.52	6.56	6.56	30	
			18.66	26.53	3.36	—	h_0	
	140	Col_h	3.08	4.34	20.39	20.39	10	*a* = 23.53
			5.33	7.51	11.78	11.77	11	
			6.16	8.69	10.19	10.20	20	
			8.16	11.51	7.69	7.71	21	
			9.25	13.06	6.79	6.80	30	
			18.34	26.07	3.42	—	h_0	
	280	Col_h	3.03	4.27	20.73	20.73	10	*a* = 23.94
			5.24	7.39	11.98	11.97	11	
			6.06	8.54	10.36	10.37	20	
			8.01	11.29	7.84	7.83	21	
			9.09	12.78	6.91	6.91	30	
			18.03	25.65	3.48	—	h_0	
***S*-HPt**	25	Col_h	3.18	4.49	19.75	19.75	10	*a* = 22.75
			5.52	7.78	11.38	11.40	11	
			6.38	9.01	9.84	9.88	20	
			8.44	11.93	7.44	7.46	21	
			9.57	13.52	6.56	6.58	30	
			18.63	26.49	3.37	—	h_0	
	140	Col_h	3.11	4.38	20.19	20.19	10	*a* = 23.33
			5.39	7.59	11.65	11.66	11	
			6.22	8.75	10.11	10.10	20	
			8.23	11.63	7.63	7.63	21	
			9.31	13.14	6.75	6.73	30	
			18.42	26.19	3.41	—	h_0	
	280	Col_h	3.02	4.26	20.79	20.79	10	*a* = 23.94
			5.24	7.39	11.98	12.00	11	
			6.06	8.54	10.36	10.4	20	
			8.02	11.33	7.83	7.86	21	
			9.11	12.86	6.89	6.93	30	
			18.04	25.72	3.48	—	h_0	

[a] Data from XRD measurements on cooling process. [b] Miller indices. [c] Lattice constants.

7. Chiroptical spectra of *R*/*S*-HPt

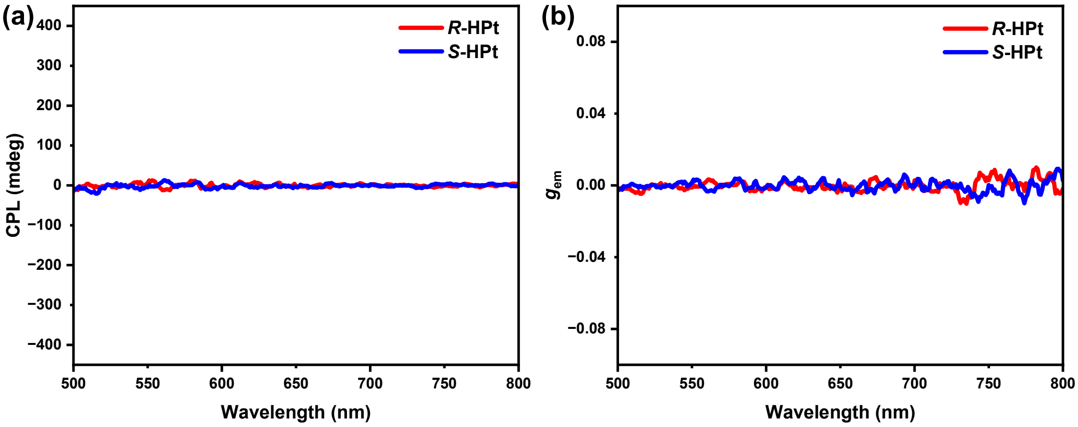

Fig. S18 Chiroptical spectra of ***R*/*S*-HPt** before thermal annealing (ATA) treatment.

8. SEM images of complexes *R*/*S*-HPt

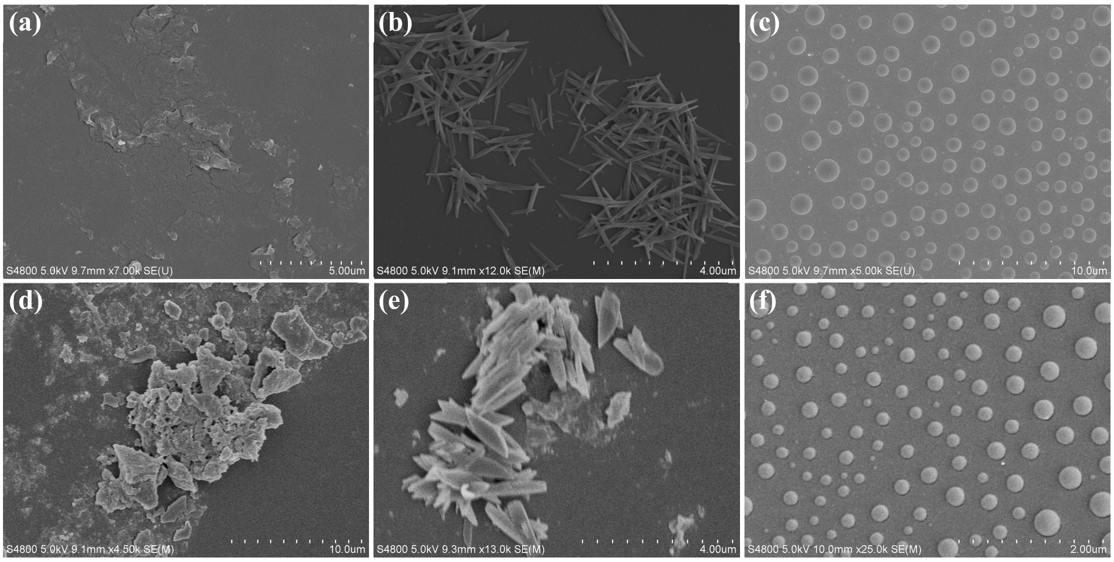

Fig. S19 SEM images of chiral self-assemblies ***R*-HPt** (a–c) and ***S*-HPt** (d–f) before thermal annealing treatment. (a) and (d): film, 1.0 × 10^{-3} mol L^{-1} in chloroform; (b) and (e): film, 1.0 × 10^{-4} mol L^{-1} in chloroform; (c) and (f): aggregated state, 1.0 × 10^{-4} mol L^{-1} in THF/H_2O = 80/20 (v/v).

9. MD simulations data of *S*-HPt-C4

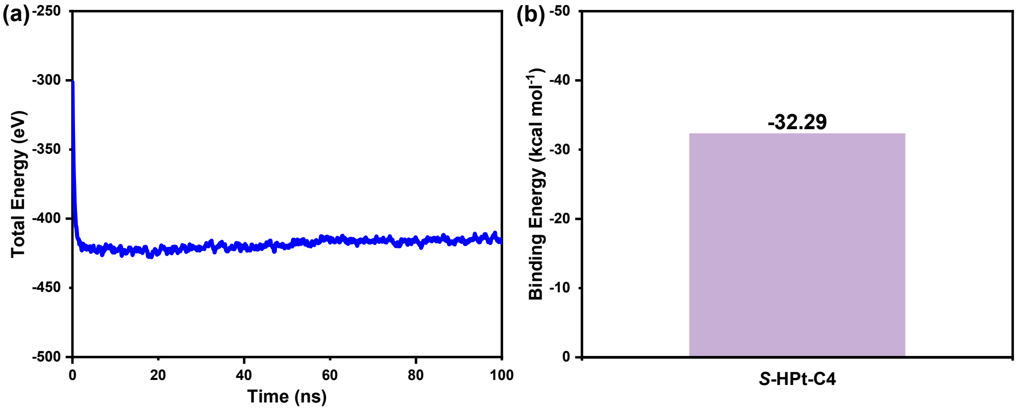

Fig. S20 The total energy (a) and binding energy (b) of self-assemblies ***S*-HPt-C4**.

10. Cyclic voltammetry (CV) curves of complexes *R*/*S*-HPt

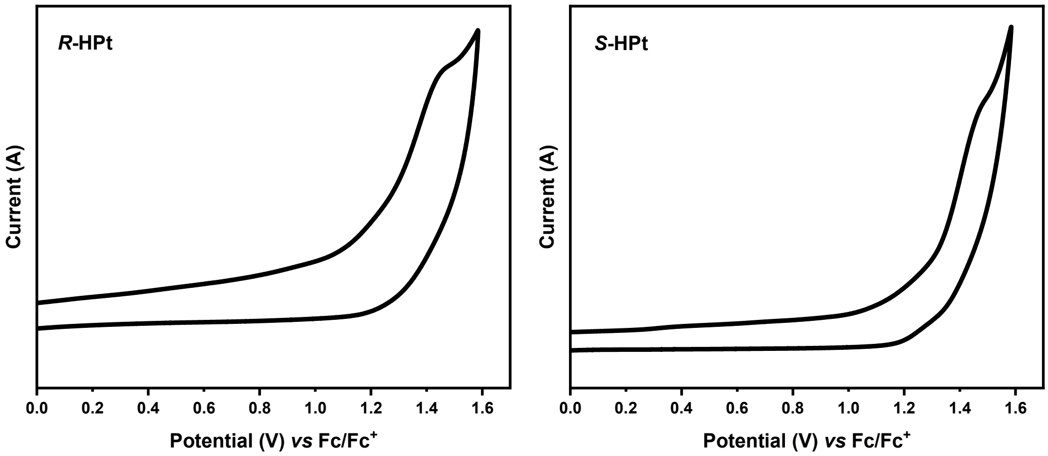

Fig. S21 CV curves of complex ***R*/*S*-HPt**. CV data were measured in degassed CH_2Cl_2 solution ($M = 1.0 \times 10^{-3}$ mol/L) with Pt wire, Ag/AgCl electrode and glassy carbon as counter, reference, and working electrodes, respectively. The scan rate was 100 mV/s, and ferrocene was used as a reference.

Table S4 The CV data of ***R*/*S*-HPt**.

	E_g (eV)	E_{HOMO} (eV)	E_{LUMO} (eV)
***R*-HPt**	3.10	-5.68	-2.58
***S*-HPt**	3.10	-5.71	-2.61

$E_g = 1240/\lambda_{edge}$ (data calculated from the absorption band edge of the solution); $E_{HOMO} = -(E_{ox} + 4.8)$ eV; $E_{LUMO} = E_g + E_{HOMO}$.

11. EL performance of homoleptic complex-based devices

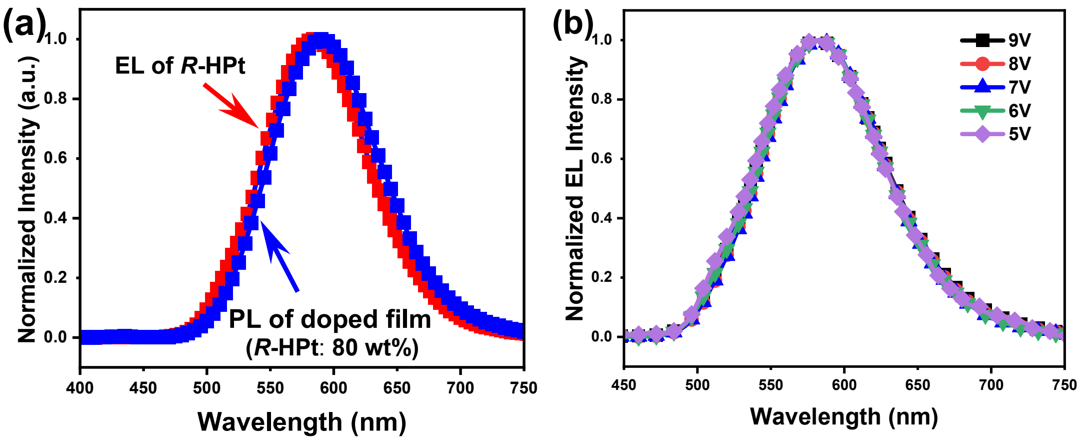

Fig. S22 (a) The EL and PL spectra of ***R*-HPt**-based CP-EL device and doped film; (b) the EL spectra of ***S*-HPt** at different driving voltages.

Table S5 Key performance data of ***R*/*S*-HPt**-based CP-OLEDs.

Device	V_{on} (V)	λ_{EL} (nm)	L_{max} (cd m^{-2})	CE_{max} (cd A^{-1})	EQE (%)	g_{EL}	CIE (x, y)
***R*-HPt**	3.7	584	10184	3.26	1.14	0.012	(0.50, 0.49)
***S*-HPt**	3.6	584	11379	3.30	1.12	−0.014	(0.50, 0.49)

V_{on}: the turn-on voltage at a brightness of 1 cd m^{-2}; g_{EL}: measured at λ_{EL} = 584 nm.

Table S6 Comparison of the luminance (L_{max}) and electroluminescence dissymmetry factor (g_{EL}) values for Pt(II) complex-based CP-EL devices.

complex	λ_{EL} (nm)	L_{max} (cd m^{-2})	g_{EL}	Reference
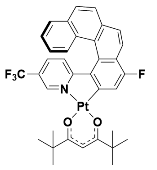	612	8424	-1.6×10^{-3}	[24]
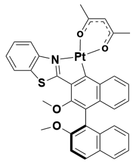	600	1062	1.20×10^{-3}	[25]
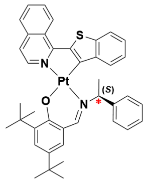	732	0.007 W sr^{-1} m^{-2}	-2.73×10^{-3}	[26]
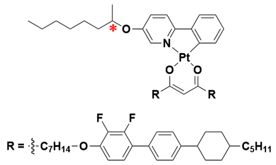	~538	7150	6.0×10^{-2}	[18]
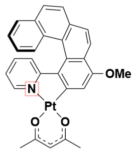	615	374	0.38	[27]
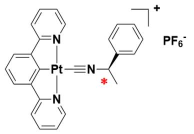	412	637	10^{-4}	[28]
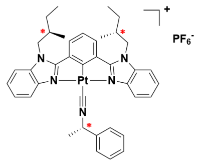	504<br>544<br>586	1693	2.6×10^{-3}	[29]

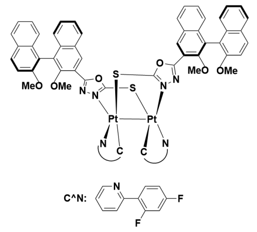	608	16106	-2.0×10^{-3}	[30]
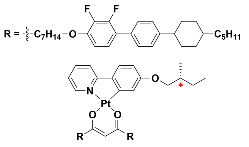	503<br>542	2087	5.0×10^{-3}	[31]
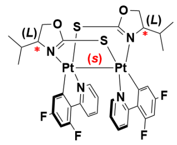	610	3955	-2.81×10^{-3}	[32]
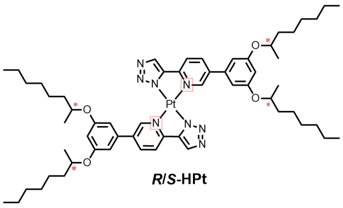 ***R*/*S*-HPt**	584	11379	-0.014	This work

12. MALDI-TOF MS spectra of homoleptic Pt(II) complexes *R*/*S*-HPt

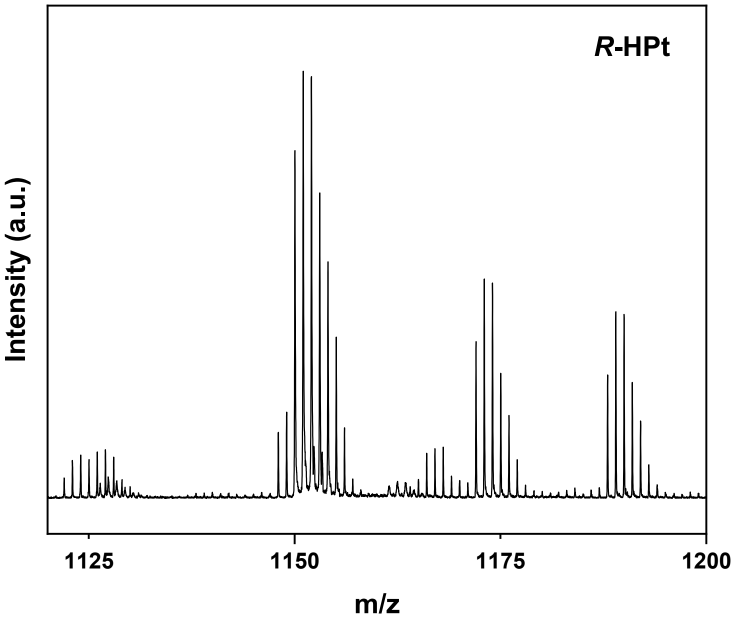

Fig. S23 MALDI-TOF mass spectrum of complex ***R*-HPt**.

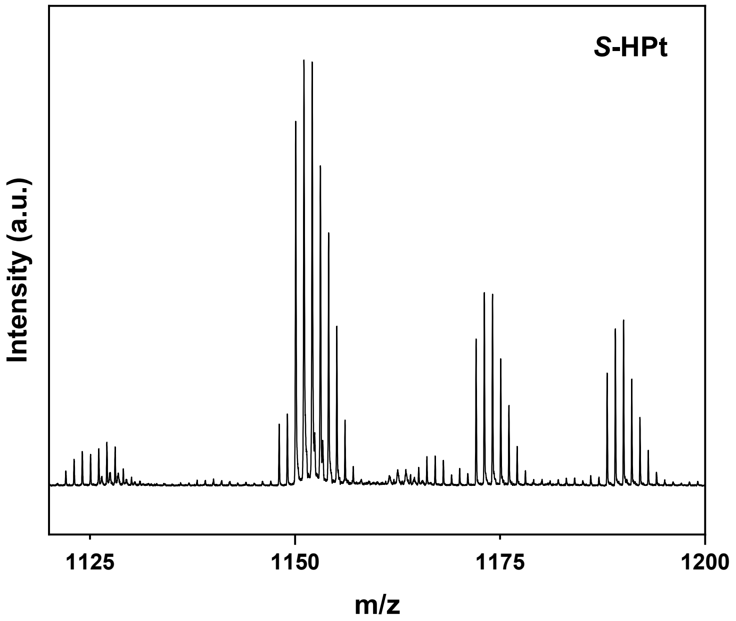

Fig. S24 MALDI-TOF mass spectrum of complex ***S*-HPt**.

13. NMR spectra of homoleptic Pt(II) complexes *R*/*S*-HPt

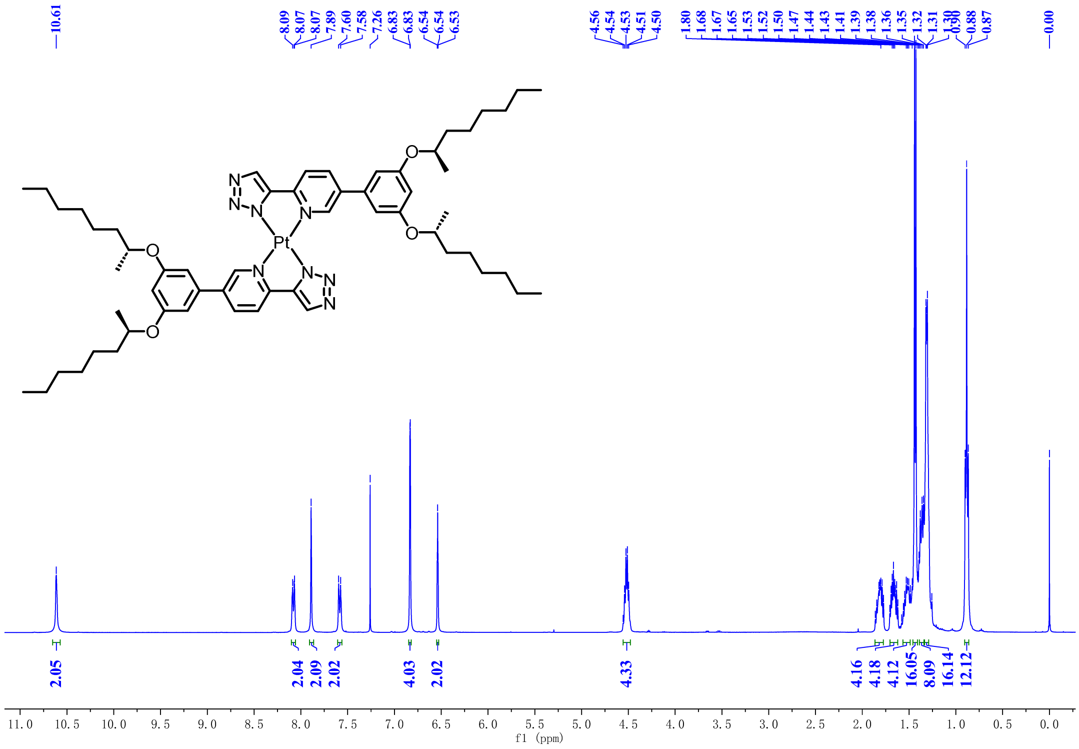

Fig. S25 ^{1}H NMR (500 MHz, $CDCl_3$) spectrum of complex ***R*-HPt**.

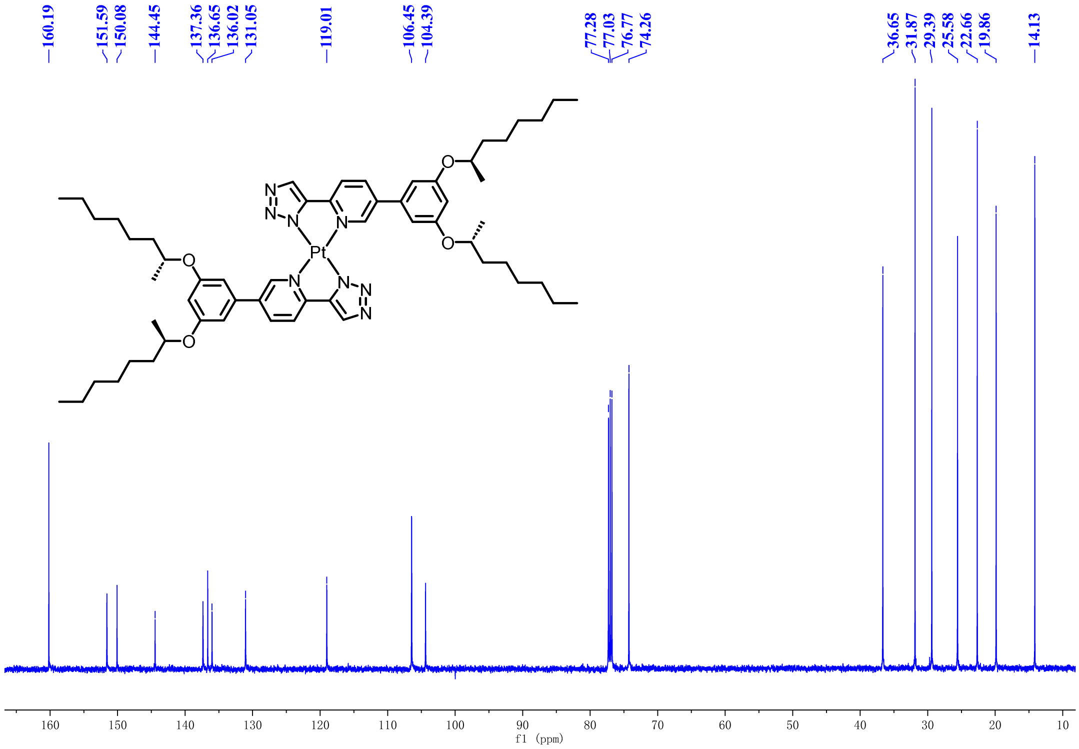

Fig. S26 ^{13}C NMR (126 MHz, $CDCl_3$) spectrum of complex ***R*-HPt**.

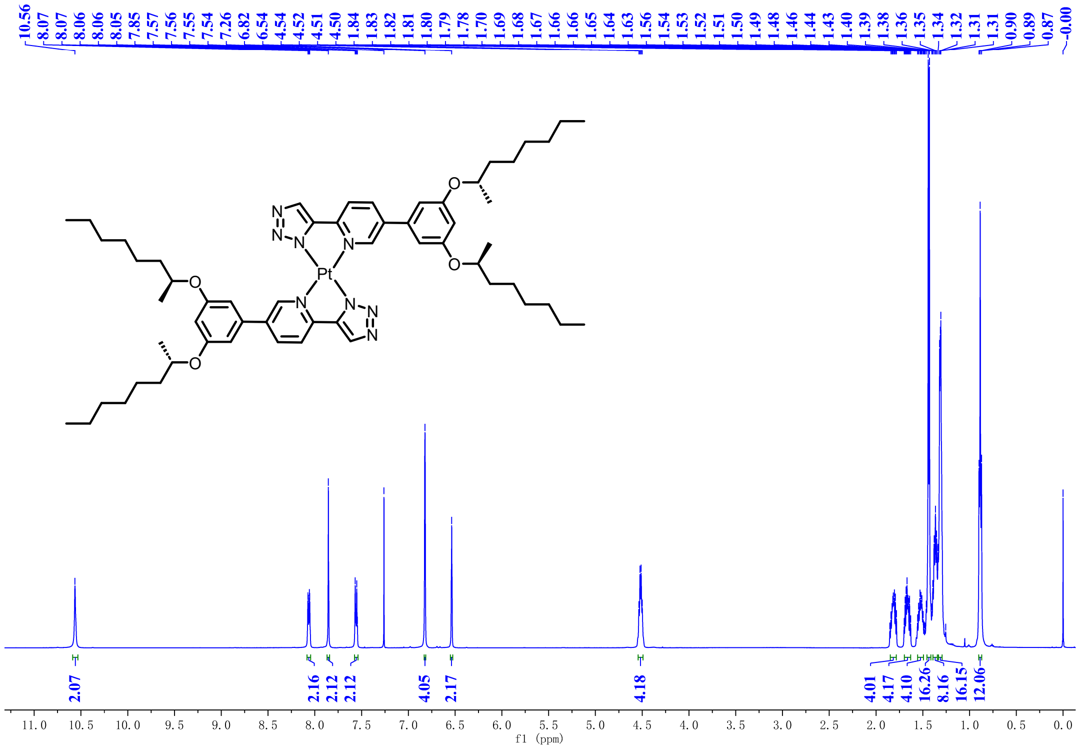

Fig. S27 ^{1}H NMR (500 MHz, $CDCl_3$) spectrum of complex ***S*-HPt**.

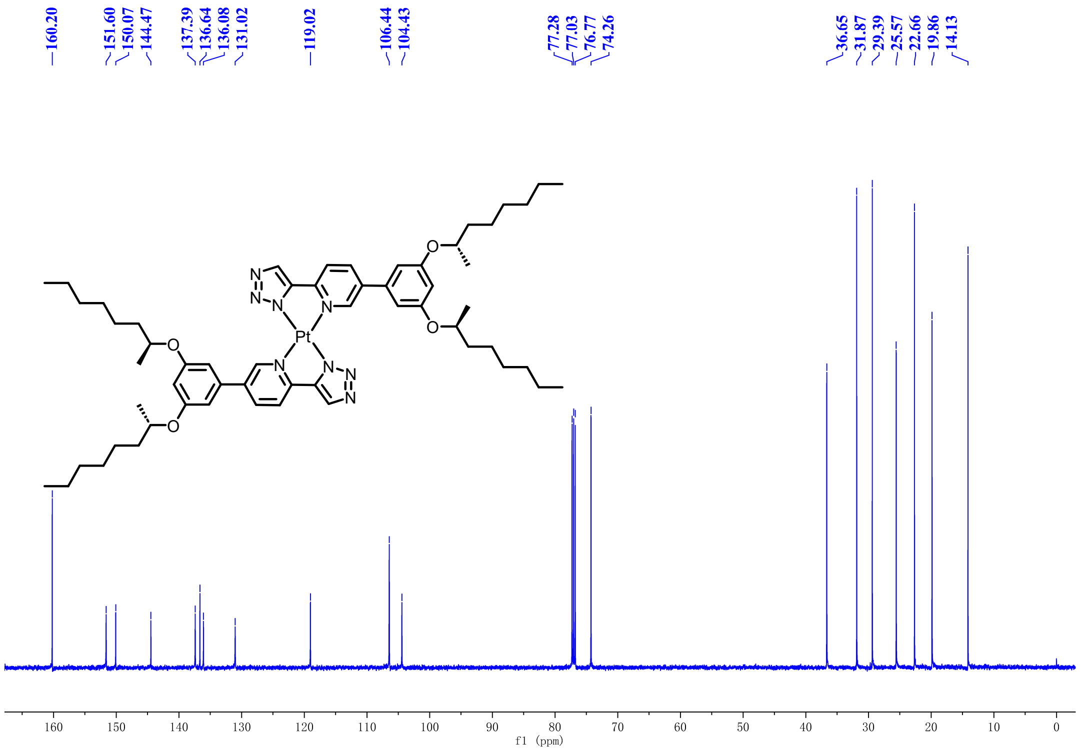

Fig. S28 ^{13}C NMR (126 MHz, $CDCl_3$) spectrum of complex ***S*-HPt**.

14. HPLC spectra of homoleptic Pt(II) complexes *R*/*S*-HPt

<Chiral Chromatography Report of *R*-HPt>

Column	CHIRALPAK IA (IA00CE-QE006)
Column size	4.6 mm ϕ × 250 mm
Injection	10.0 µL
Mobile phase	Hexane/THF = 80/20 (V/V)
Flow rate	1.0 mL/min
Wavelength	300 nm
Temperature	40 ºC
HPLC equipment	Shimadzu SPD-20A; Daicel Chiralpak IA Column
Sample	*R*-HPt

<Chromatogram>

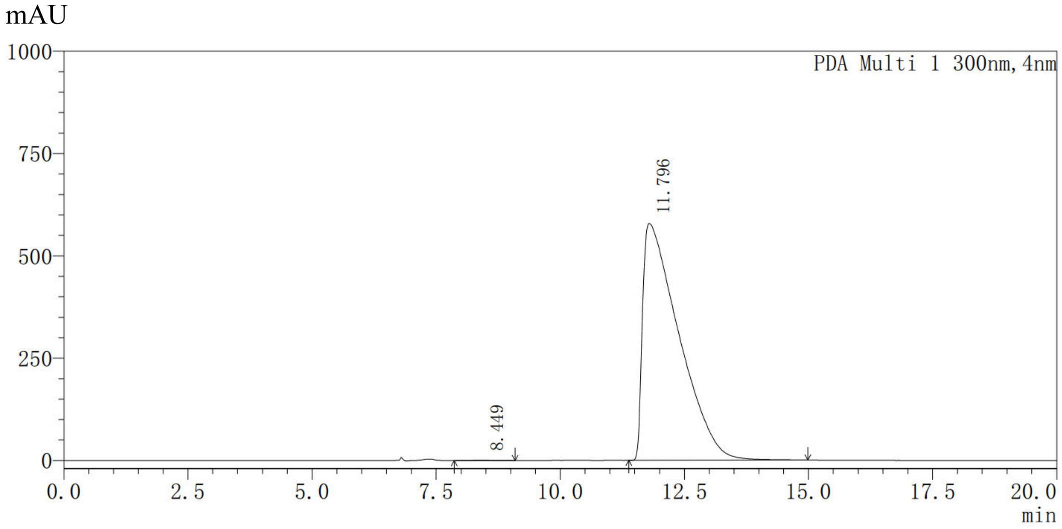

Fig. S29 HPLC spectrum of homoleptic Pt(II) complex ***R*-HPt**.

<Peak Table>

PDA Ch1 300 nm

Peak#	Start Time	End Time	Ret. Time	Area	Area%
1	7.861	9.088	8.449	9372	0.032
2	11.381	14.987	11.796	29403477	99.968
Total				29412849	100.000

<Chiral Chromatography Report of *S*-HPt>

Column	CHIRALPAK IA (IA00CE-QE006)
Column size	4.6 mm ϕ × 250 mm
Injection	10.0 μL
Mobile phase	Hexane/THF = 80/20 (V/V)
Flow rate	1.0 mL/min
Wavelength	300 nm
Temperature	40 ºC
HPLC equipment	Shimadzu SPD-20A; Daicel Chiralpak IA Column
Sample	*S*-HPt

<Chromatogram>

mAU

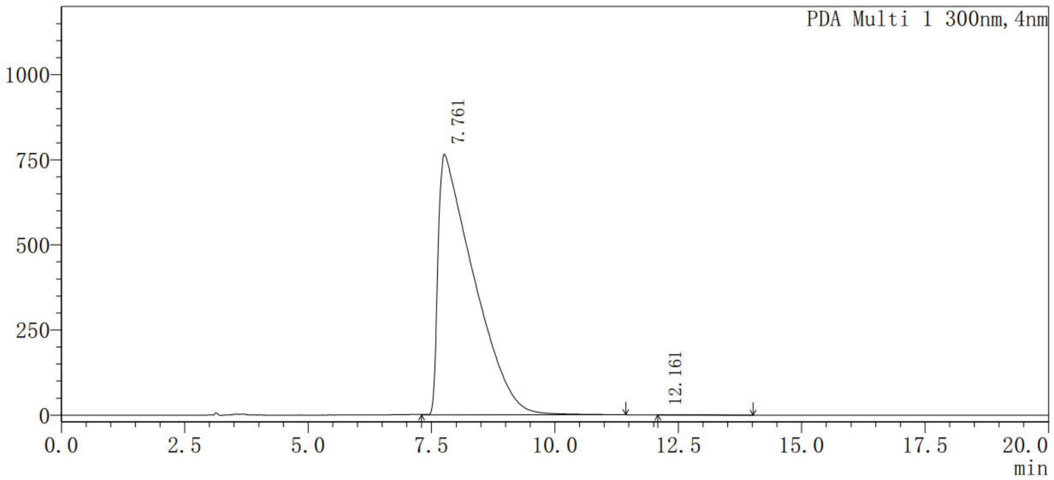

Fig. S30 HPLC spectrum of homoleptic Pt(II) complex ***S*-HPt**.

<Peak Table>

PDA Ch1 300 nm

Peak#	Start Time	End Time	Ret. Time	Area	Area%
1	7.296	11.435	7.761	38713308	99.991
2	12.085	14.016	12.161	3390	0.009
Total				38716699	100.000

15. References

[1] Tuesuwan, B.; Kerwin, S. M. *Biochemistry-US* **2006**, *45*, 7265-7276.

[2] Ishiyama, T.; Murata, M.; Miyaura, N. *J. Org. Chem.* **1995**, *60*, 7508-7510.

[3] Sharpless, K. B.; Loren, J. C.; Krasiński, A.; Fokin, V. V. *Synlett* **2005**, *9*, 2847-2850.

[4] Frisch, M. J. T., G. W.; Schlegel, H. B.; Scuseria, G. E.; Robb, M. A.; Cheeseman, J. R.; Scalmani, G.; Barone, V.; Petersson, G. A.; Nakatsuji, H.; Li, X.; Caricato, M.; Marenich, A. V.; Bloino, J.; Janesko, B. G.; Gomperts, R.; Mennucci, B.; Hratchian, H. P.; Ortiz, J. V.; Izmaylov, A. F.; Sonnenberg, J. L.; Williams, Ding, F.; Lipparini, F.; Egidi, F.; Goings, J.; Peng, B.; Petrone, A.; Henderson, T.; Ranasinghe, D.; Zakrzewski, V. G.; Gao, J.; Rega, N.; Zheng, G.; Liang, W.; Hada, M.; Ehara, M.; Toyota, K.; Fukuda, R.; Hasegawa, J.; Ishida, M.; Nakajima, T.; Honda, Y.; Kitao, O.; Nakai, H.; Vreven, T.; Throssell, K.; Montgomery Jr., J. A.; Peralta, J. E.; Ogliaro, F.; Bearpark, M. J.; Heyd, J. J.; Brothers, E. N.; Kudin, K. N.; Staroverov, V. N.; Keith, T. A.; Kobayashi, R.; Normand, J.; Raghavachari, K.; Rendell, A. P.; Burant, J. C.; Iyengar, S. S.; Tomasi, J.; Cossi, M.; Millam, J. M.; Klene, M.; Adamo, C.; Cammi, R.; Ochterski, J. W.; Martin, R. L.; Morokuma, K.; Farkas, O.; Foresman, J. B.; Fox, D. J. *Gaussian 16, Revision B.01 Gaussian, Inc., Wallingford CT,: 2016*,

[5] Stephens, P. J.; Devlin, F. J.; Chabalowski, C. F.; Frisch, M. J. *J. Phys. Chem.* **1994**, *98*, 11623-11627.

[6] Marenich, A. V.; Cramer, C. J.; Truhlar, D. G. *J. Phys. Chem. B* **2009**, *113*, 6378-6396.

[7] Lu, T.; Chen, F. *J. Comput. Chem.* **2012**, *33*, 580-592.

[8] Grimme, S.; Antony, J.; Ehrlich, S.; Krieg, H. *J. Chem. Phys.* **2010**, *132*, 154104.

[9] Johnson, E. R.; Becke, A. D. *J. Chem. Phys.* **2005**, *123*, 024101.

[10] Becke, A. D.; Johnson, E. R. *J. Chem. Phys.* **2005**, *123*, 154101.

[11] Johnson, E. R.; Becke, A. D. *J. Chem. Phys.* **2006**, *124*, 174104.

[12] Grimme, S.; Ehrlich, S.; Goerigk, L. *J. Comput. Chem.* **2011**, *32*, 1456-1465.

[13] Abraham, M. J.; Murtola, T.; Schulz, R.; Páll, S.; Smith, J. C.; Hess, B.; Lindahl, E. *SoftwareX* **2015**, *1-2*, 19-25.

[14] Lindorff-Larsen, K.; Piana, S.; Palmo, K.; Maragakis, P.; Klepeis, J. L.; Dror, R. O.; Shaw, D. E. *Proteins: Struct., Funct., Bioinf.* **2010**, *78*, 1950-1958.

[15] Martínez, L.; Andrade, R.; Birgin, E. G.; Martínez, J. M. *J. Comput. Chem.* **2009**, *30*, 2157-2164.

[16] Berendsen, H. J. C.; Postma, J. P. M.; van Gunsteren, W. F.; DiNola, A.; Haak, J. R. *J. Chem. Phys.* **1984**, *81*, 3684-3690.

[17] van Duijneveldt, F. B.; van Duijneveldt-van de Rijdt, J. G. C. M.; van Lenthe, J. H. *Chem. Rev.* **1994**, *94*, 1873-1885.

[18] Qian, G.; Yang, X.; Wang, X.; Herod, J. D.; Bruce, D. W.; Wang, S.; Zhu, W.; Duan, P.; Wang, Y. *Adv. Opt. Mater.* **2020**, *8*, 2000775.

[19] Prabhath, M. R. R.; Romanova, J.; Curry, R. J.; Silva, S. R. P.; Jarowski, P. D. *Angew. Chem. Int. Ed.* **2015**, *54*, 7949-7953.

[20] Zou, G.; Zhao, L.; Zeng, L.; Luo, K.; Ni, H.; Wang, H.; Li, Q.; Yu, W.; Li, X. *Inorg. Chem.* **2019**, *58*, 861-869.

[21] Wu, J.; Watson, M. D.; Zhang, L.; Wang, Z.; Müllen, K. *J. Am. Chem. Soc.* **2004**, *126*, 177-186.

[22] Yasuda, T.; Shimizu, T.; Liu, F.; Ungar, G.; Kato, T. *J. Am. Chem. Soc.* **2011**, *133*, 13437-13444.

[23] Jasiński, M.; Szymańska, K.; Gardias, A.; Pociecha, D.; Monobe, H.; Szczytko, J.; Kaszyński, P. *ChemPhysChem* **2019**, *20*, 636-644.

[24] Yan, Z.-P.; Luo, X.-F.; Liu, W.-Q.; Wu, Z.-G.; Liang, X.; Liao, K.; Wang, Y.; Zheng, Y.-X.; Zhou, L.; Zuo, J.-L.; Pan, Y.; Zhang, H. *Chem. Eur. J.* **2019**, *25*, 5672-5676.

[25] Jiang, Z.; Wang, J.; Gao, T.; Ma, J.; Liu, Z.; Chen, R. *ACS Appl. Mater. Interfaces* **2020**, *12*, 9520-9527.
[26] Fu, G.; He, Y.; Li, W.; Wang, B.; Lü, X.; He, H.; Wong, W.-Y. *J. Mater. Chem. C* **2019**, *7*, 13743-13747.
[27] Brandt, J. R.; Wang, X.; Yang, Y.; Campbell, A. J.; Fuchter, M. J. *J. Am. Chem. Soc.* **2016**, *138*, 9743-9746.
[28] Han, J.; Lu, H.; Xu, Y.; Guo, S.; Zheng, X.; Tao, P.; Liu, S.; Zhang, X.; Zhao, Q. *J. Organomet. Chem.* **2020**, *915*, 121240.
[29] Han, J.; Wang, Y.; Wang, J.; Wu, C.; Zhang, X.; Yin, X. *J. Organomet. Chem.* **2022**, *973-974*, 122394.
[30] Song, J.; Xiao, H.; Fang, L.; Qu, L.; Zhou, X.; Xu, Z.-X.; Yang, C.; Xiang, H. *J. Am. Chem. Soc.* **2022**, *144*, 2233-2244.
[31] Fan, P.; Fang, Z.; Wang, S.; Dong, Q.; Xiao, C.; McEllin, A. J.; Bruce, D. W.; Zhu, W.; Wang, Y. *Chin. Chem. Lett.* **2023**, *34*, 107934.
[32] Song, J.; Xiao, H.; Zhang, B.; Qu, L.; Zhou, X.; Hu, P.; Xu, Z.-X.; Xiang, H. *Angew. Chem. Int. Ed.* **2023**, *62*, e202302011.
